# A wild rice-derived peptide R14 ameliorates monosodium urate crystals-induced IL-1β secretion through inhibition of NF-κB signaling and NLRP3 inflammasome activation

**DOI:** 10.7717/peerj.15295

**Published:** 2023-05-12

**Authors:** Supattra Charoenwutthikun, Kasem Chanjitwiriya, Sittiruk Roytrakul, Duangkamol Kunthalert

**Affiliations:** 1 Department of Microbiology and Parasitology, Faculty of Medical Science, Naresuan University, Phitsanulok, Thailand; 2 National Center for Genetic Engineering and Biotechnology, National Science and Technology Development Agency, Thailand Science Park, Pathumthani, Thailand; 3 Centre of Excellence in Medical Biotechnology, Faculty of Medical Science, Naresuan University, Phitsanulok, Thailand

**Keywords:** Gout, Monosodium urate, Interleukin-1β, NF-κB, Inflammasome

## Abstract

Gout is an inflammatory arthritis initiated by the deposition of monosodium urate crystals (MSU) around the joints and surrounding tissues. MSU crystals activate the nucleotide-binding oligomerization domain-like receptor containing pyrin domain 3 (NLRP3) inflammasome to the release of interleukin-1β (IL-1β). Gout can have a substantial impact on patient’s quality of life, and currently available medicines are unable to meet all the clinical needs. This study explored anti-gout potentials of the Rice14 (R14) peptide, a peptide derived from leaves of wild rice *Oryza minuta*. The effects of R14 peptide on IL-1β secretion in THP-1 macrophages with MSU crystals-induced inflammation were examined. Our results clearly showed that the R14 peptide significantly inhibited the secretion of IL-1β in MSU crystals-induced macrophages, and the effects were dose-related. For safety testing, the R14 peptide did not show both cytotoxicity and hemolytic activity. In addition, the R14 peptide strongly suppressed the phospho-IκB-α and nuclear factor kappa-B (NF-κB) p65 proteins in NF-κB signaling pathway, reduced the NLRP3 expression and inhibited the MSU crystals-mediated cleavage of caspase-1 as well as mature IL-1β. The R14 peptide also reduced MSU-triggered intracellular ROS levels in macrophages. Taken together, these results indicated that R14 peptide inhibited MSU crystals-induced IL-1β production through NF-κB and NLRP3 inflammasome activation. Our findings demonstrated that R14 peptide, the newly recognized peptide from wild rice, possessed potent regulatory activity against IL-1β production in MSU crystals-induced inflammation, and we therefore propose that the R14 peptide is a promising molecule with potential clinical application in the treatment of MSU crystals-induced inflammation.

## Introduction

Gout is the most common and complex form of inflammatory arthritis and its prevalence and incidence appear to be rising across the globe (Dehlin, Jacobsson & Roddy, 2020; Singh & Gaffo, 2020). Gout is caused by hyperuricemia that leads to the formation and deposition of monosodium urate (MSU) crystals in joints, tendons and other tissues (So & Martinon, 2017). Clinically, gout is characterized by recurrent episodes of pronounced acute inflammation, usually affecting a single joint. In the long-term hyperuricemia, the disease typically progresses to the formation of MSU crystal deposits (tophi) in soft tissues, recurrent attacks of arthritis affecting multiple joints and progressive joint destruction (So & Martinon, 2017). Additionally, gout is often accompanied by various comorbidities, including cardiovascular disease, hypertension and chronic kidney disease (Singh & Gaffo, 2020), and this further complicates clinical management and disease outcomes. Nowadays, the treatment of gout attack depends on drugs such as nonsteroidal anti-inflammatory drugs (NSAIDs), glucocorticoids, colchicine, and IL-1β antagonists (Burns & Wortmann, 2011). The inevitable side effects such as gastrointestinal toxicity, renal toxicity and gastrointestinal bleeding, however, limits their clinical uses (Bensman, 2020; Slobodnick et al., 2018; Stewart et al., 2020). The quest for an alternative drug with desirable efficacy and acceptable safety profile therefore remains an important focus.

Gout is now considered as an inflammatory disease driven by activation of the innate immune system. MSU crystals, the major etiological agent of gout, stimulate macrophages to release interleukin-1β (IL-1β), which is mediated by nucleotide-binding oligomerization domain-like receptor containing pyrin domain 3 (NLRP3) inflammasome activation (Kingsbury, Conaghan & McDermott, 2011; Martinon et al., 2006). The NLRP3 inflammasome is a multiprotein complex consisting of the NLRP3 domain, an apoptosis-associated speck like protein containing a caspase recruitment domain (ASC) and a caspase-1 domain (Swanson, Deng & Ting, 2019). Activation of the NLRP3 inflammasome involves a two-step process. Priming (signal 1) is mediated through the nuclear factor-kappa B (NF-κB) pathway, leading to the upregulation of pro-IL-1β and NLRP3 protein levels. MSU crystals provide the second signal (signal 2), triggering the assembly of the NLRP3 inflammasome (Yang et al., 2020). MSU crystal induces the NLRP3 inflammasome through sensing of decreases intracellular potassium ion concentrations due to increased intracellular Na^+^, water influx and cellular swelling (Schorn et al., 2011), and the production of reactive oxygen species (ROS) (Zhou et al., 2011). ROS that is generated by MSU crystals has also been shown to be a potential mediator in inflammasome activation (Cao et al., 2023; Wang et al., 2021; Zhou et al., 2011). Once activated, the NLRP3 inflammasome promotes the proteolytic cleavage of the precursor of IL-1β (pro-IL-1β) into its mature biologically active form (Martinon & Tschopp, 2007). Significantly, the massive and uncontrolled release of IL-1β is deemed as an initiation event that induces gouty inflammation and promotes the recruitment of vast numbers of neutrophils at the sites of inflammation (Martinon, 2010a; Mitroulis, Kambas & Ritis, 2013). Studies so far have firmly established that IL-1β plays an essential role in the pathogenesis of gouty inflammation (Dinarello, 2005; Kingsbury, Conaghan & McDermott, 2011; Martinon & Tschopp, 2004; Reber et al., 2014). Increasing evidence have also supported the involvement of IL-1β in gout progression and severity (Crişan et al., 2016; Dinarello, 2010; Torres et al., 2009). As for the importance of IL-1β in gouty inflammation whose production is mediated in an inflammasome-dependent manner, targeting IL-1β as well as the components in inflammasome pathway may provide promising avenues for therapeutic treatment of gout.

Bioactive peptides, generally 2-20 amino acids in length, represent a unique class of compounds with special physiological functions affecting the cardiovascular, endocrine, immune, and nervous systems. Numerous studies have reported that bioactive peptides are of great value for physiological function regulation, including anti-oxidation, anti-hypertension, anti-thrombosis, anti-microbial properties, anti-cancer, anti-inflammation, anti-diabetic, anti-obesity, cholesterol-lowering and immunomodulatory activities (Daliri, Oh & Lee, 2017; Jia et al., 2021; Pavlicevic, Marmiroli & Maestri, 2022). Besides their biological activities, bioactive peptides are also recognized for being highly selective and efficacious and, at the same time, relatively safe and well tolerated (Fosgerau & Hoffmann, 2015). Consequently, there is an increased interest in development of the bioactive peptides as therapeutics. A number of active peptides have been globally approved for a wide range of diseases, including diabetes, cancer, osteoporosis, multiple sclerosis, HIV infection and chronic pain (Muttenthaler et al., 2021; Wang et al., 2022). Insulin, exenatide, liraglutide, enfuvirtide and ziconotide are examples of peptide drugs that have been in clinical uses (Muttenthaler et al., 2021; Wang et al., 2022). Nevertheless, research and discovery of bioactive peptides that can effectively inhibit MSU crystals-induced inflammation in gout remain scarce.

In the recent years, peptides derived from rice protein or their hydrolysates have attracted increasing attention. The protein isolates from rice are highly nutritious and similar to casein and soy protein isolates (Jayaprakash et al., 2022; Wang et al., 2018). In addition, their nutritional quality, digestibility, and hypo-allergenicity makes rice protein an alternative source of protein over animal-based and gluten-containing protein (Jayaprakash et al., 2022; Wang et al., 2018). In the previous study, protein hydrolysates from 13 wild rice species were prepared and assessed for their biological activities (Moung-ngam et al., 2020). Among which, protein hydrolysates from wild rice *Oryza minuta* leaves showed the greatest biological activities including growth inhibition against cancer cells (Moung-ngam et al., 2020). Such protein hydrolysates were subsequently subjected to chromatographic purification and amino acid sequencing, 30 peptide candidates were selected for peptide synthesis and anti-cancer activities determined (Moung-ngam et al., 2020). In parallel, the immunomodulatory activities of those peptide candidates were also examined. Our preliminary study suggested the immunomodulatory potential of the Rice14 (R14) peptide. Since the development of new anti-gout agents remain an important focus, this study therefore explored anti-gout potentials of the Rice14 (R14) peptide, a synthetic peptide derived from leaves of wild rice *O. minuta*. We assessed the inhibitory effects of the R14 peptide in MSU crystals-induced inflammation, with particular emphasized on the production of IL-1β, the master cytokine in gout inflammation. The possible mechanisms responsible for its action were also investigated.

## Material and Methods

### Reagents and antibodies

Monosodium urate (MSU) crystals were obtained from InvivoGen (Carlsbad, CA, USA). Phorbol 12-myristate 13-acetate (PMA), colchicine, 3-(4,5-dimethylthiazol-2-yl)-2,5-diphenyltetrazolium bromide (MTT) and dimethyl sulfoxide (DMSO; ≥99.5%) were bought from Sigma-Aldrich (St. Louis, MO, USA). Antibodies for IL-1β (12242) and NLRP3 (15101) were purchased from Cell Signaling Technology (USA). Antibodies against caspase-1 (sc-56036), phospho-IκB-α (sc-52943) and NF-κB p65 (sc-8008) were from Santa Cruz Biotechnology (Dallas, TX, USA) and β-actin antibody was from Abcam (Cambridge, UK). Peroxidase-conjugated AffiniPure goat anti-mouse IgG (115-035-003), and anti-rabbit IgG HRP-linked antibody (7074) as secondary antibodies were obtained from Jackson ImmunoResearch Laboratories (West Grove, PA, USA) and Cell Signaling Technology, respectively.

### Peptide

Rice14 (R14) peptide at the purity of ≥ 95.0% (by HPLC) was synthesized by GenScript (Piscataway, NJ, USA). The amino acid sequence of R14 peptide was ILIILDD and its molecular weight was 813.98 Da. The test peptide was dissolved in its vehicle, DMSO and further diluted in culture medium to achieve desired concentrations.

### Hemolytic activity

The hemolytic activity of R14 peptide was examined according to a protocol described previously (Lee et al., 2004). Briefly, suspension of 2% sheep red blood cells (100 µL) prepared in phosphate buffered saline (PBS) pH 7.4 was incubated with 100 µL of R14 peptide at the concentrations ranged from 5 µM to 100 µM. After incubation at 37 °C for 1 h, the suspension was centrifuged at 1,000 g for 5 min and the supernatant transferred to 96 well-microtiter plate (Nunc™, Roskilde, Denmark). Released hemoglobin was then determined by measuring an absorbance at 405 nm using a microplate reader (Molecular Devices, San Jose, CA, USA). Triton X-100 (1%) and PBS pH 7.4 served as positive and negative controls, respectively. The percentage of hemolysis was calculated using an equation: (OD405 nm peptide – OD405 nm PBS pH 7.4)/(OD405 nm 1% Triton X-100 – OD405 nm PBS pH 7.4) ×100.

### Cell culture and THP-1 differentiation

Human monocytic THP-1 cells (TIB-202) were purchased from American Type Culture Collection (ATCC; Manassas, VA, USA). THP-1 cells were cultured in RPMI 1640 medium (HyClone™ Logan, Utah, USA) supplemented with 10% (v/v) heat-inactivated fetal bovine serum (Gibco, Billings, MT, USA), 2 mM L-glutamine (Gibco, Billings, MT, USA), 100 U/mL penicillin, 100 µg/mL streptomycin (Gibco, USA), 10 mM 4-(2-hydroxyethyl)-1 piperazineethanesulfonic acid (HEPES; HyClone™) and 0.05 mM 2-mercaptoethanol (Bio-Rad, Hercules, CA, USA). Cells were maintained in a humidified incubator at 37 °C with 5% CO_2_ and sub-cultured every 2–3 days.

To differentiate THP-1 cells into macrophages, the THP-1 cells at a density of 1 ×10^5^ cells/well in 96-well tissue culture plates (Nunc™) or 2 ×10^6^ cells/well in 6-well plates (Nunc™) were treated with 50 ng/mL PMA for 24 h at 37 °C in 5% CO_2_. Cells were then washed twice with PBS pH 7.4 and maintained in antibiotic- and serum-free RPMI 1640 medium for 24 h at 37 °C in 5% CO_2_ and used in subsequent experiments.

### Cell viability assay

The cytotoxic effect of R14 peptide was evaluated by MTT assay according to a method previously described (Mosmann, 1983). Differentiated THP-1 cells at the density of 1 ×10^5^ cells/well were incubated with R14 peptide at the concentrations varied from 5 µM to 100 µM, or vehicle. Cells without the test peptide served as an untreated control. After 24, 48 and 72h-incubation at 37 °C in 5% CO_2_, 20 µL MTT solution (5 mg/mL) was added to each well and incubated for additional 3 h at 37 °C in 5% CO_2_. After the removal of supernatant, 100 µL of DMSO was added to dissolve the formazan crystals. The plates were shaken vigorously to ensure complete solubilization. The optical density at 540 nm was determined using a microplate reader (Molecular Devices, San Jose, CA, USA). The percentage of cell viability was calculated based on the equation: (OD of treated cells/ OD of untreated cells) ×100.

### Quantification of IL-1β levels

Differentiated THP-1 cells (1 ×10^5^ cells/well) were incubated with MSU crystals (100 µg/mL) in the presence or absence of various concentrations of R14 peptide (5–100 µM) for 6 h at 37 °C in 5% CO_2_. Subsequently, the supernatants were collected, and the levels of IL–1β were quantified by sandwich enzyme-linked immunosorbent assay (ELISA) using ELISA MAX™ Deluxe Set (BioLegend, San Diego, CA, USA), according to the manufacturer’s standard protocols. The detection limit for the IL-1β was 0.5 pg/mL.

### Western blotting

Differentiated THP-1 cells (2 ×10^6^ cells/well) were incubated with MSU crystals in the presence or absence of various concentrations of R14 peptide (5–100 µM) for 30 min (for p-IκB-α and NF-κB p65) or 6 h (for caspase-1, IL-1β and NLRP3) at 37 °C in 5% CO_2_. Supernatants were collected and cell pellets were washed with cold PBS pH 7.4, followed by protein extraction and Western blot analysis as a protocol reported previously (Chanjitwiriya, Roytrakul & Kunthalert, 2020). Nuclear and cytoplasmic proteins were extracted according to a previously described method (Jantaruk et al., 2017). The concentrations of proteins extracted were determined using a Bradford protein assay kit (Bio-Rad). Extracted proteins were suspended with Laemmli sample buffer (Bio-Rad), heated at 95 °C for 5 min and subsequently separated by SDS-polyacrylamide gel electrophoresis (SDS-PAGE) using 10% TGX™ FastCast™ Acrylamide gel (Bio-Rad). Afterwards, resolved proteins were transferred to nitrocellulose membranes (Bio-Rad) with semi-dry transfer system (Bio-Rad). After being blocked by incubation with 5% skim milk in TTBS (20 mM Tris, 150 mM NaCl, 0.1% Tween20) for 1 h at room temperature, the membranes were washed three times with TTBS and then incubated overnight with primary antibodies specific for p-IκB-α, NF-κB p65, caspase-1, IL-1β, NLRP3 and β-actin. After rinsing twice with TTBS, the membranes were reacted with peroxidase-conjugated AffiniPure goat anti-mouse IgG or anti-rabbit IgG, HRP-linked antibody, as appropriate, for 1 h at room temperature. The protein bands were visualized by Clarity™ Western ECL Substrate (Bio-Rad) according to the manufacturer’s instruction and captured using an ImageQuant LAS 4000 Biomolecular Imager (GE Healthcare Life Sciences, Chicago, IL, USA). The relative intensities of the protein bands were measured by ImageJ software and then normalized against the internal control β-actin.

### Measurement of intracellular ROS generation

Intracellular ROS measurement was performed using DCFDA / H2DCFDA-Cellular Reactive Oxygen Species Detection Assay Kit (Abcam, Cambridge, UK), according to the manufacturer’s standard protocols. Briefly, differentiated THP-1 cells (1 ×10^5^ cells/well) were pre-treated with DCFDA solution (20 µM) for 45 min and further stimulated with MSU crystals (100 µg/mL) in the presence or absence of various concentrations of R14 peptide (5–100 µM) for 6 h at 37 °C in 5% CO_2_. Cells were washed twice with PBS pH 7.4 and fluorescence intensity was then monitored at an excitation wavelength at 485 nm and an emission wavelength at 535 nm using a microplate reader (BioTek Synergy HT, Winooski, VT, USA).

### Statistical analysis

Data are presented as mean ± standard deviation (SD) of at least three independent experiments. Difference between test and control was analyzed by two-tailed student’s *t*-test using IBM SPSS Statistics for Windows, Version 26.0 (Armonk, NY, USA). A *p-*value of less than 0.05 was considered to be statistically significant.

## Results

### Effects of R14 peptide on cell viability

**Figure 1:**
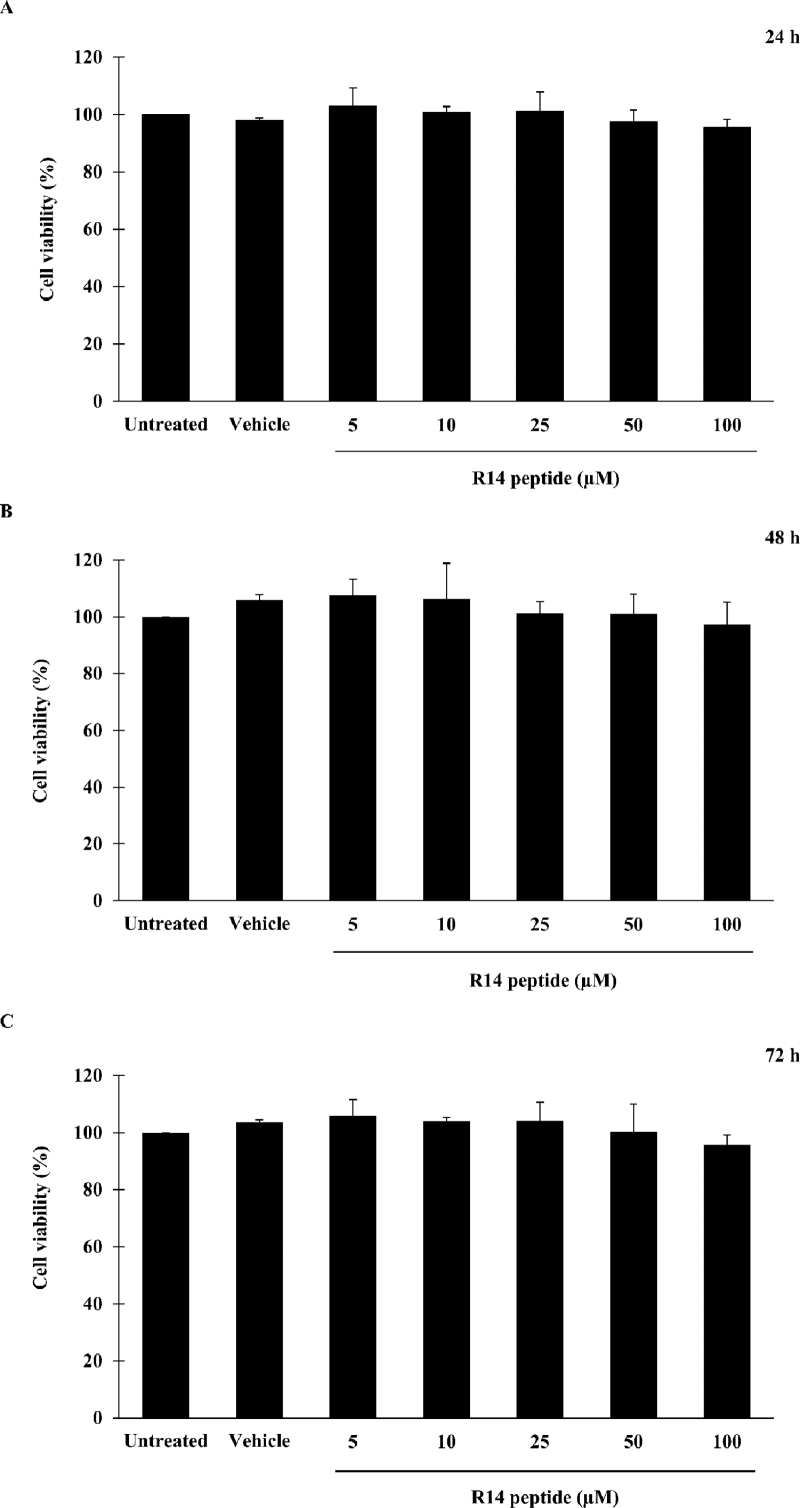
R14 peptide showed no cytotoxicity in THP-1 macrophages after 24, 48 and 72 h incubation. THP-1 macrophages were treated with R14 peptide at concentrations ranged from 5–100 µM, or vehicle for 24 h (A), 48 h (B), and 72 h (C), and cell viability was determined by an MTT assay. Cells without the R14 peptide served as an untreated control. Values are expressed as mean ± SD of three independent experiments.

Prior to investigating the inhibitory activity of R14 peptide, its cytotoxicity against THP-1 macrophages was examined by an MTT assay. As shown in Fig. 1, viability of THP-1 macrophages exposed to R14 peptide at 24, 48 and 72 h was not significantly changed at any of the peptide concentrations compared with the untreated control. MTT assay also showed no significant difference in cell viability between vehicle and untreated control. The results indicated that the R14 peptide under the concentrations examined when exposure time increases as well as the vehicle were not toxic to THP-1 macrophages.

### Effects of R14 peptide on hemolysis

To further examine the toxicity of R14 peptide, hemolytic activity was conducted. As presented in Table 1, it was found that incubation of sheep red blood cells with R14 peptide showed no such activity, suggesting that the R14 peptide had no toxic effects on red blood cells.

**Table 1:** Hemolytic activity of R14 peptide.

Treatment	% Hemolysis^a^
Untreated control	0.00 ± 0.00
Triton X-100	100.00 ± 0.00
Vehicle control	0.02 ± 0.03
R14 peptide, 5 µM	0.00 ± 0.00
R14 peptide, 10 µM	0.10 ± 0.16
R14 peptide, 25 µM	0.07 ± 0.12
R14 peptide, 50 µM	0.03 ± 0.05
R14 peptide, 100 µM	0.06 ± 0.10

**Notes.**

a The results represent the mean ± SD of three independent experiments.

### Effects of R14 peptide on MSU crystals-induced IL-1β secretion in THP-1 macrophages

**Figure 2:**
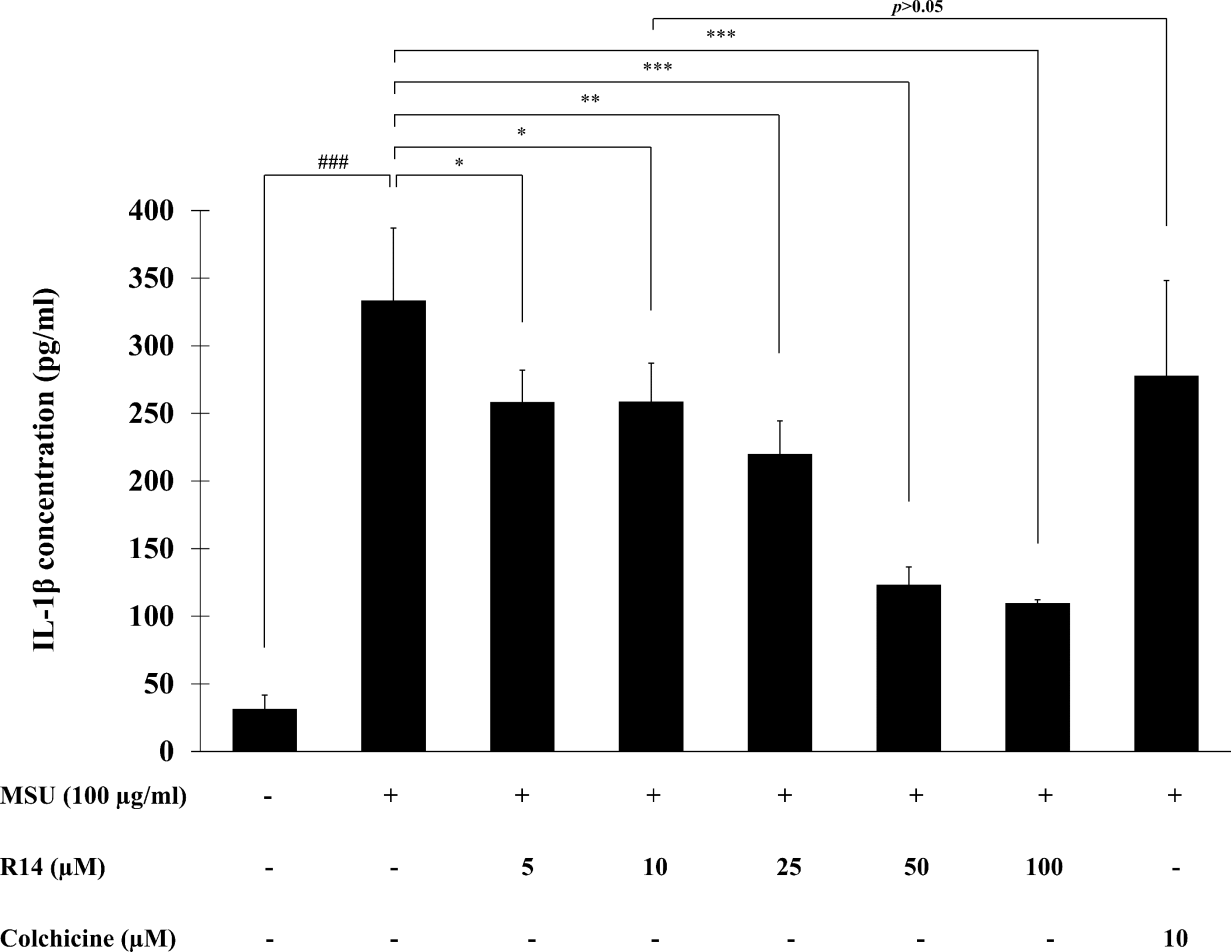
R14 peptide inhibited the IL-1β production in MSU crystals-induced THP-1 macrophages. THP-1 macrophages were stimulated with MSU crystals (100 µg/mL) in the presence or absence of R14 peptide (5–100 µM) or colchicine (10 µM) for 6 h. The levels of IL-1β production were determined by sandwich ELISA. Values are expressed as mean ± SD of at least three independent experiments. ### *p* < 0.001 compared to the unstimulated THP-1 macrophages. * *p* < 0.05, ** *p* < 0.01 and *** *p* < 0.001 compared with the MSU crystal-induced THP-1 macrophages.

To investigate the inhibitory activity of R14 peptide on MSU crystals-induced IL-1β secretion in THP-1 macrophages, THP-1 cells stimulated with MSU crystals were incubated with the R14 peptide (5–100 µM) for 6 h and the IL-1β level in culture supernatant quantified by ELISA. The results presented in Fig. 2 showed that stimulation of MSU crystals dramatically enhanced the production of IL-1β in THP-1 macrophages as compared with the untreated control. Treatment with the R14 peptide however produced a marked decrease in the elevated levels of IL-1β in THP-1 macrophages. Such reduction by the R14 peptide appeared to be concentration-related and this was significantly observed from 5–100 µM, with % inhibition ranged from 20.30–66.48%. It is interesting to note that R14 peptide at 10 µM inhibited IL-1β production in a similar level (*p* > 0.05) to that of the reference drug, colchicine (10 µM). No inhibitory effect of colchicine at 5 µM was observed under our experimental settings (data not shown).

### Effects of R14 peptide on the activation of NLRP3 inflammasome and IL-1β synthesis in MSU crystals-induced THP-1 macrophages

**Figure 3:**
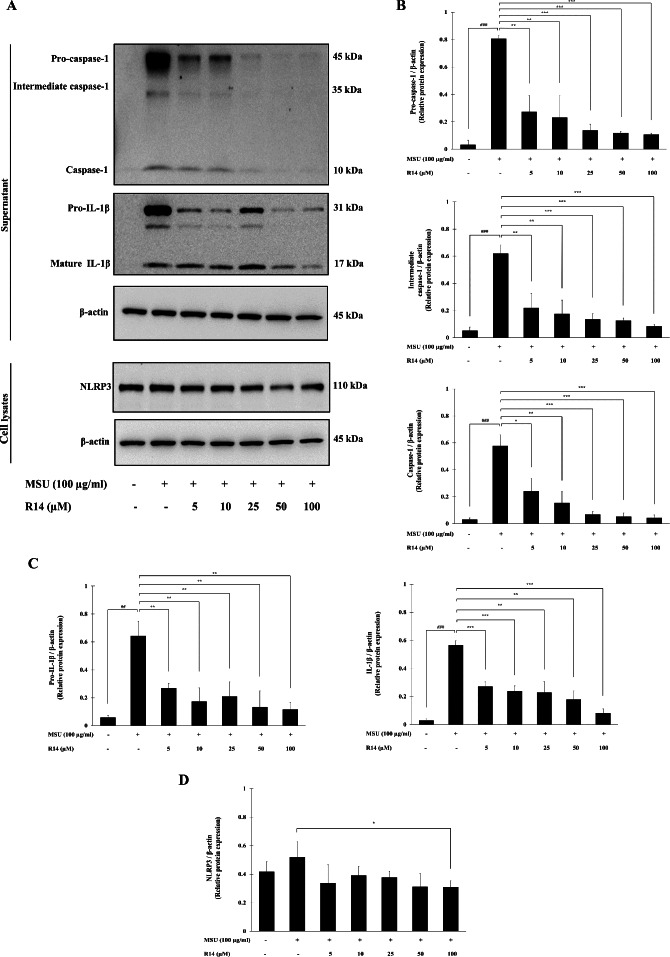
R14 peptide inhibited the activation of NLRP3 inflammasome and IL-1β synthesis in MSU crystals-induced THP-1 macrophages. THP-1 macrophages were stimulated with MSU crystals (100 µg/mL) in the presence or absence of R14 peptide (5–100 µM). After 6 h incubation, the protein expressions from supernatants and cell lysates were examined by Western blotting. The Western blot images of protein bands are the representative of three separate experiments (A). Bar diagrams showing densitometric analysis of the relative expression of pro-caspase-1/β-actin, intermediate caspase-1/β-actin, caspase-1/β-actin (B), pro-IL-1β/β-actin, IL-1β/β-actin (C) and NLRP3/β-actin (D), quantified using ImageJ software. Values are expressed as mean ± SD of three independent experiments. ## *p* < 0.01, ### *p* < 0.001 compared to the unstimulated THP-1 macrophages. * *p* < 0.05, ** *p* < 0.01 and *** *p* < 0.001 compared with the MSU crystals-induced THP-1 macrophages.

To verify whether the inhibition of IL-1β secretion by R14 peptide contributed to the inhibition of NLRP3 inflammasome activation, Western blotting to detect crucial inflammasome-related proteins was conducted. The results in Fig. 3 demonstrated that the expression of pro-caspase-1, intermediate caspase-1 and caspase-1 was markedly increased in THP-1 macrophages stimulated with MSU crystals, which was dose-dependently suppressed by the treatment of R14 peptide (Figs. 3A and 3B). Meanwhile, marked increases in the expression of pro-IL-1β, and mature IL-1β were observed, which was also significantly decreased in the presence of R14 peptide (Figs. 3A and 3C). Moreover, significant reduction in the NLRP3 protein expression was observed by R14 treatment, in particular at the concentration of 100 µM (Figs. 3A and 3D).

### Effects of R14 peptide on MSU crystals-induced activation of NF-κB signaling pathway in THP-1 macrophages

**Figure 4:**
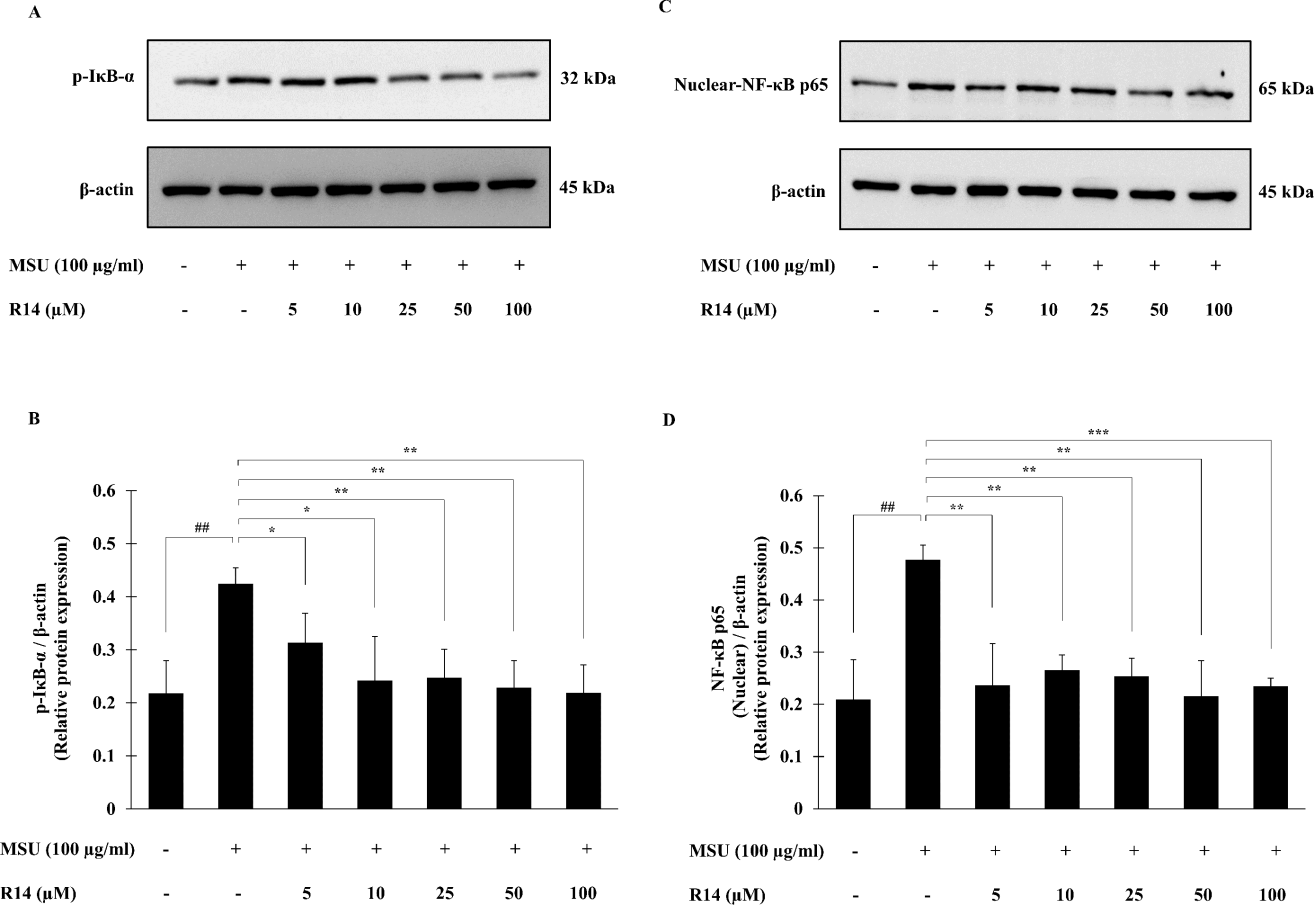
R14 peptide suppressed the NF-*κ*B activation in MSU crystals-induced THP-1 macrophages. THP-1 macrophages were stimulated with MSU crystals (100 µg/mL) in the presence or absence of R14 peptide (5–100 µM). After 30 min incubation, the expression of p-IκB-α and nuclear-NF-κB p65 protein were determined by Western blotting. The Western blot images of protein bands are the representative of three separate experiments. Bar diagrams showing densitometric analysis of the relative expression of p-IκB-α/β-actin (B) and nuclear NF-κB p65/β-actin (D), quantified using ImageJ software. Values are expressed as mean ± SD of three independent experiments. ## *p* < 0.01 compared to the unstimulated THP-1 macrophages. * *p* < 0.05, ** *p* < 0.01 and *** *p* < 0.001 compared with the MSU crystals-induced THP-1 macrophages.

The activation of NF-κB signaling pathway plays a pivotal role in inflammatory response and NLRP3 inflammasome activation (Liu et al., 2017; Mahmoud et al., 2019). To investigate whether the R14 peptide induced suppression of NLRP3 inflammasome activation depends on the NF-κB pathway, expression of the major components of NF-κB activation, the p-IκB-α as well as nuclear-NF-κB p65 was determined. Western blot analysis showed that the expression of p-IκB-α protein was significantly elevated in THP-1 macrophages following MSU stimulation, and this was reduced by the treatment of the R14 peptide (Figs. 4A and 4B). Also, the expression of nuclear-NF-κB p65 protein was significantly increased in THP-1 macrophages stimulated with MSU crystals. In the presence of R14 peptide, however, significant decrease in nuclear-NF-κB p65 expression was evident compared with MSU-treated group without the test peptide (Figs. 4C and 4D). Of note, the decreases in both p-IκB-α and nuclear-NF-κB p65 protein expression by the R14 peptide were close to the baseline seen in the unstimulated control.

### Effect of R14 peptide on MSU crystals-induced ROS production in THP-1 macrophages

**Figure 5:**
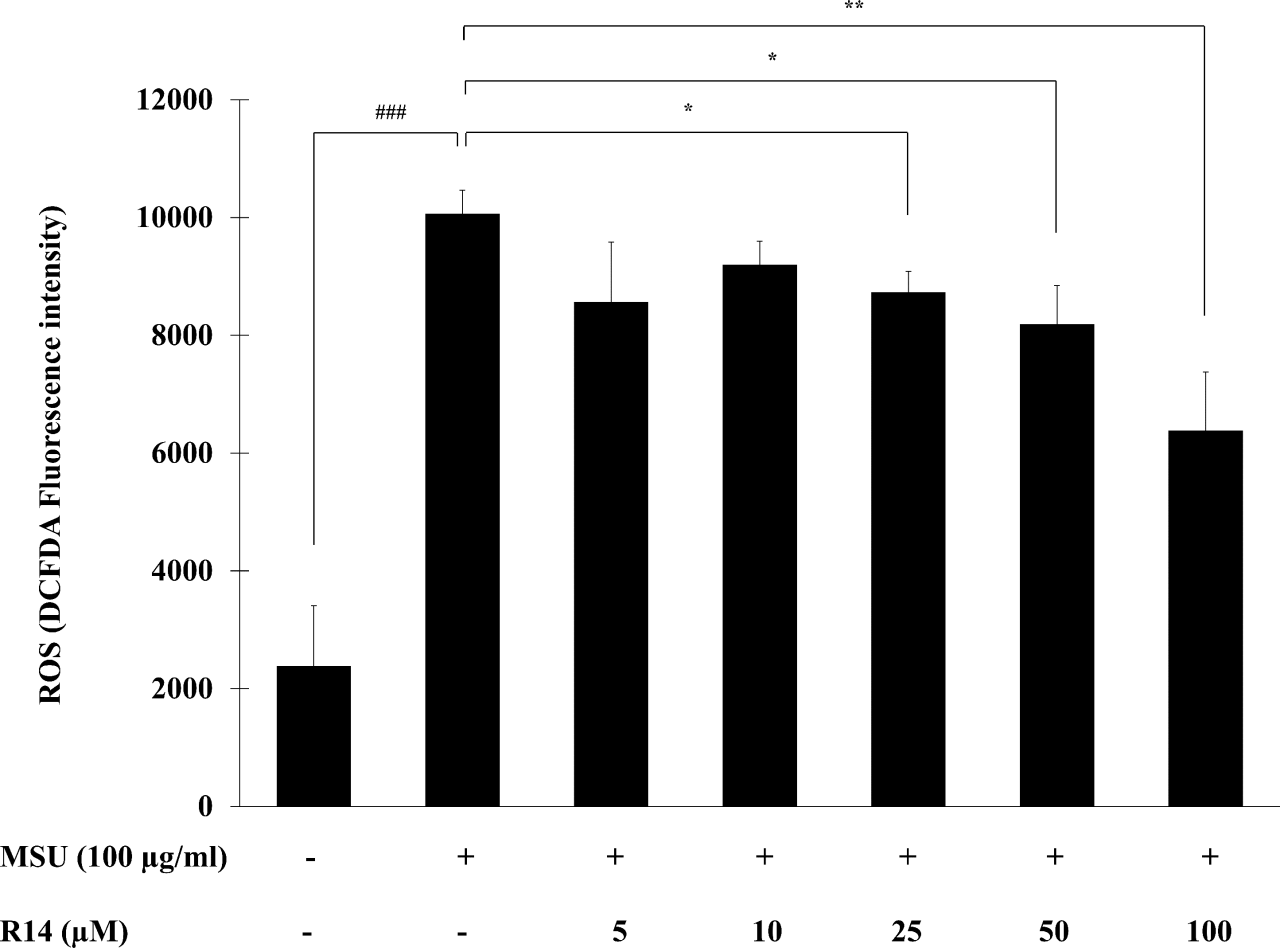
R14 peptide reduced the intracellular ROS generation in MSU crystals-induced THP-1 macrophages. THP-1 macrophages were stimulated with MSU crystals (100 µg/mL) in the presence or absence of R14 peptide (5–100 µM) for 6 h. The levels of ROS production were determined by measuring fluorescence at an excitation wavelength at 485 nm and an emission wavelength at 535 nm. Values are expressed as mean ± SD of three independent experiments. ### *p* < 0.001 compared to the unstimulated THP-1 macrophages. * *p* < 0.05 and ** *p* < 0.01 compared with the MSU crystal-induced THP-1 macrophages.

The ROS play a critical role in activation of the NLRP3 inflammasome as a second activation signal (Jo et al., 2016; Martinon, 2010b), we further examined whether the R14 peptide could reduce the oxidative stress in MSU crystals-induced THP-1 macrophages. The results in Fig. 5 showed that MSU crystals obviously induced ROS production in THP-1 macrophages, as indicated by an increase in intracellular DCFDA fluorescence intensity. However, treatment with the R14 peptide significantly reduced MSU crystals-triggered ROS generation in THP-1 macrophages, compared to MSU crystals stimulation alone. The effect was evident at the peptide concentrations ranging from 25 to 100 µM.

## Discussion

It is well documented that MSU crystals stimulate the synthesis and release of IL-1β and that IL-1β plays a critical role in gout inflammation (Dinarello, 2005; Kingsbury, Conaghan & McDermott, 2011; Martinon & Tschopp, 2004; Reber et al., 2014). In an attempt to search for new therapeutics for treatment of gout attacks, we investigated the regulatory potentials of the wild rice-derived peptide, R14 on MSU crystals-induced secretion of IL-1β in macrophage cells. Our results showed that the R14 peptide effectively reduced IL-1β secretion induced by MSU crystals in a dose-dependent manner. Such the reduction was not due to direct cellular cytotoxicity since MTT assay showed no significant changes in macrophage cell viability upon exposure to the R14 peptide. In terms of % inhibition, the R14 peptide inhibited the MSU crystals-induced production of IL-1β in a similar level to that of colchicine, the reference drug, suggesting their efficacies were comparable. Although colchicine is a widely used and recommended first line therapy for the treament of gout, undesirable adverse effects have however been reported, including severe diarrhea, vomiting and nausea (Slobodnick et al., 2018; Stewart et al., 2020). Additionally, long-term use of colchicine may also lead to serious adverse effects such as renal impairment, myopathy, and rhabdomyolysis (Fernández-Cuadros et al., 2019; Richette, Frazier & Bardin, 2014). The fact that the R14 peptide exhibited no hemolytic activity toward normal erythrocytes, this suggested the very low toxicity to the mammalian cells and would surpass the limitation for development of this peptide as future therapeutics. Our findings therefore demonstrated that the R14 peptide is a promising therapeutic agent and warrant further development of this peptide for control the MSU crystals-mediated inflammation in gout.

In gout, the mature and functional IL-1β production triggered by MSU crystals requires two prerequisite steps, priming and activation (Guo, Callaway & Ting, 2015; So & Martinon, 2017). Priming (signal 1) is mediated by NF-κB activating pathway, in which a member of the Toll-like receptor (TLR) family have been implicated in MSU crystal’s identification and activation (Crişan et al., 2016; Liu et al., 2019). This signalling cascade induces the expression of NLRP3 inflammasome and IL-1β precursor (pro-IL-1β) (Guo, Callaway & Ting, 2015; Mariotte et al., 2020). MSU crystals provide signal 2 and initiate the assembly of the inflammasome protein complex. The assembled NLRP3 inflammasome leads to the activation of caspase-1, which autocleaves pro-caspase-1 into active caspase-1 and eventually cleaves pro-IL-1β to generate mature IL-1β (Kingsbury, Conaghan & McDermott, 2011). Previous studies have demonstrated that macrophages from mice deficient in various components of the inflammasome such as caspase-1, ASC and NALP3 are defective in MSU crystals-induced IL-1β activation, supporting the pivotal role of inflammasome in gouty inflammation (Martinon et al., 2006). To gain insight into the actions by which the R14 peptide inhibited IL-1β production in MSU crystals-induced macrophages, involvement of the R14 peptide in both inflammasome priming and activation was investigated. We observed that the R14 peptide strongly inhibited the expression of p-I κB-α and nuclear-NF-κB p65 proteins, the two critical components of NF-κB activating pathway (Fig. 4). Moreover, the R14 peptide suppressed NLRP3 and pro-IL-1β protein expression and inhibited MSU crystals-mediated cleavage of caspase-1 (Fig. 3). Consistent with this, mature IL-1β production was decreased following the treatment of R14 peptide (Fig. 3). These results suggested that the R14 peptide suppressed both of the two checkpoints of inflammasome activation. To further obtain evidence of its inhibitory mechanisms on inflammasome activation, effect on the production of ROS was investigated. Generation of ROS through MSU crystals was found to enhance NLRP3 inflammasome activation (Harijith, Ebenezer & Natarajan, 2014; Yin et al., 2020). Significant increased ROS levels in macrophages upon exposure to MSU crystals have also been reported (Sorbara & Girardin, 2011). Moreover, suppression of ROS production was shown to block the synthesis and secretion of IL-1β mediated by NLRP3 inflammasome activation (Martinon, 2010b; Zhou et al., 2010). In the present study, we observed that the R14 peptide dose-dependently suppressed MSU crystals-stimulated ROS production (Fig. 5), which corresponded to a significant decrease in NLRP3 protein expression. This suggested that the inhibition of ROS production by the R14 peptide contributed to its suppressive effect on NLRP3 inflammasome activation. Taken all together, our results indicated that the R14 peptide inhibited MSU crystals-induced IL-1β production through the suppression of NF-κB and NLRP3 inflammasome pathways. The fact that the R14 peptide was added to macrophage cells at the same time of MSU crystal stimulation and the inhibition of IL-1β production observed, it is possible that the R14 peptide binds to a receptor or may penetrate the macrophage cells and subsequently interferes with signaling molecules in the signaling pathways involved in the production of IL-1β. Whether the R14 peptide acts through a receptor binding or cell penetration cannot be revealed from our experimental settings and will need to be clarified in the future investigation. Nevertheless, as the NF-κB and NLRP3 inflammasome pathways have emerged as therapeutic targets for the treatment of gout inflammation (Guo, Callaway & Ting, 2015; So & Martinon, 2017), the observed findings therefore strengthened the potential of the R14 peptide on the regulation in MSU crystals-induced production of IL-1β, the key aspect of gouty inflammation.

## Conclusions

This study clearly demonstrated for the first time that the R14 peptide, a rice-derived peptide from *Oryza minuta* leaves, possessed potent inhibitory activity against MSU crystals-induced IL-1β production in macrophages, without exhibiting cytotoxicity. The R14 peptide inhibited MSU crystals-induced IL-1β production through the block of phosphorylation of IκB-α and p65 NF-κB activation, meanwhile suppressed the activation of NLRP3 inflammasome. We therefore propose that the R14 peptide is a promising molecule and may have potential clinical application in the treatment of MSU crystals-induced inflammation. An *in vivo* model of MSU crystals-induced inflammation to determine the efficacy as well as the safety of R14 peptide will be a future requirement to confirm its potential clinical use in treatment of gouty inflammation.

##  Supplemental Information

10.7717/peerj.15295/supp-1 Supplemental Information 1 Raw data of Figs. 1–5 and Table 1

10.7717/peerj.15295/supp-2 Supplemental Information 2 Original western blot images of Figs. 3 and 4 Each blot was captured by CCD camera twice. In column A, exposure time on the Image Quant LAS4000 was set to 1/100 s and images were captured in order that the molecular weight markers could be detected. The same blots were then captured by Image Quant LAS4000 with appropriate exposure times and the respective bands visualized (column B).

## Additional Information and Declarations

### Data Availability

The following information was supplied regarding data availability: The raw measurements are available in the Supplementary Files.
