# Supplementary material for: A wild rice-derived peptide R14 ameliorates monosodium urate crystals-induced IL-1β secretion through inhibition of NF-κB signaling and NLRP3 inflammasome activation
Source: PeerJ. 2023 May 12;11:e15295. doi: 10.7717/peerj.15295 (PMC10184658; doi:10.7717/peerj.15295)
Supplement: Supplemental Information 2 — Each blot was captured by CCD camera twice. In column A, exposure time on the Image Quant LAS4000 was set to 1/100 s and images were captured in order that the molecular weight markers could be detected. The same blots were then captured by Image Quant LAS4000 with appropriate exposure times and the respective bands visualized (column B). [file peerj-11-15295-s002.docx]

**Original Western Blot Images**

**A wild rice-derived peptide R14 ameliorates monosodium urate crystals-induced IL-1β secretion through** **inhibition of NF-κB signaling and NLRP3 inflammasome activation**

Supattra Charoenwutthikun^1^, Kasem Chanjitwiriya^1^, Sittiruk Roytrakul^2^ and Duangkamol Kunthalert^1, 3^*

^1^ Department of Microbiology and Parasitology, Faculty of Medical Science, Naresuan University, Phitsanulok, Thailand

^2^ National Center for Genetic Engineering and Biotechnology, National Science and Technology Development Agency, Thailand Science Park, Pathumthani, Thailand

^3^ Centre of Excellence in Medical Biotechnology, Faculty of Medical Science, Naresuan University, Phitsanulok, Thailand

Corresponding author:

Duangkamol Kunthalert

Email address: [duangkamolk@nu.ac.th](mailto:duangkamolk@nu.ac.th), [kunthalertd@yahoo.com](mailto:kunthalertd@yahoo.com)

**Figure 3**

**Proteins from supernatant**

| **Caspase-1** | **A: Exposure time 1/100 sec** | **B: Exposure time 600 sec** |
| --- | --- | --- |
| **Exp.1** | 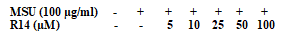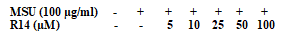 **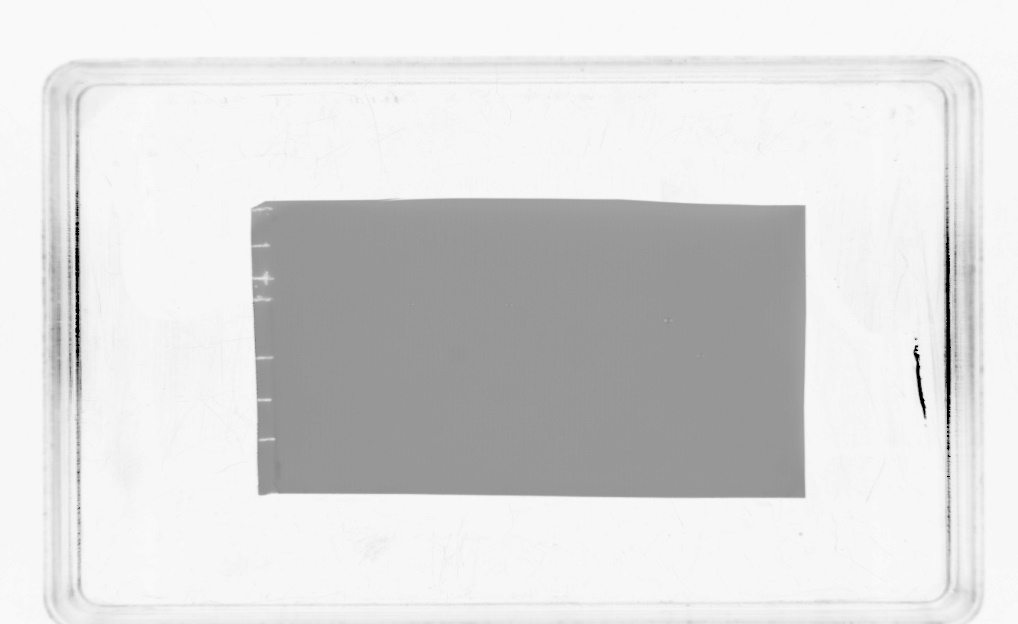**  **15**  **75**  **50**  **37**  **25**  **10**  **100** | 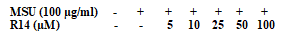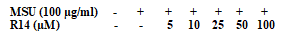 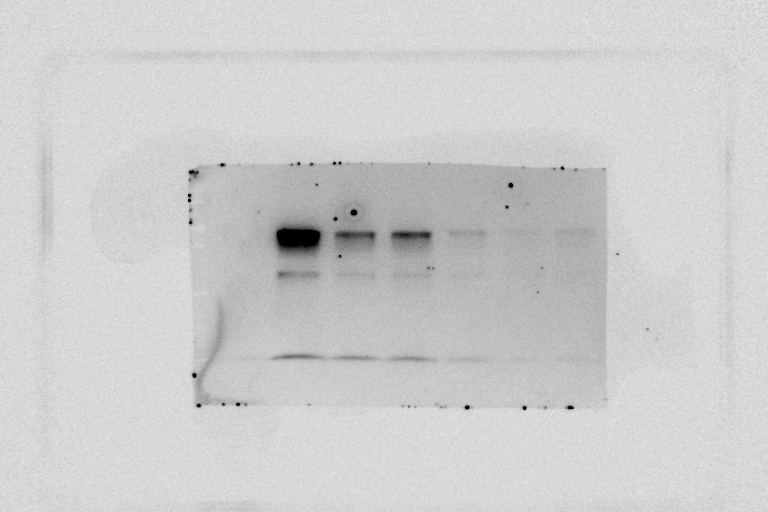  **Pro-caspase-1**  **Intermediate**  **Caspase-1**  **50**  **37**  **25**  **10**  **100**  **15**  **75** |
| **Exp.2** | **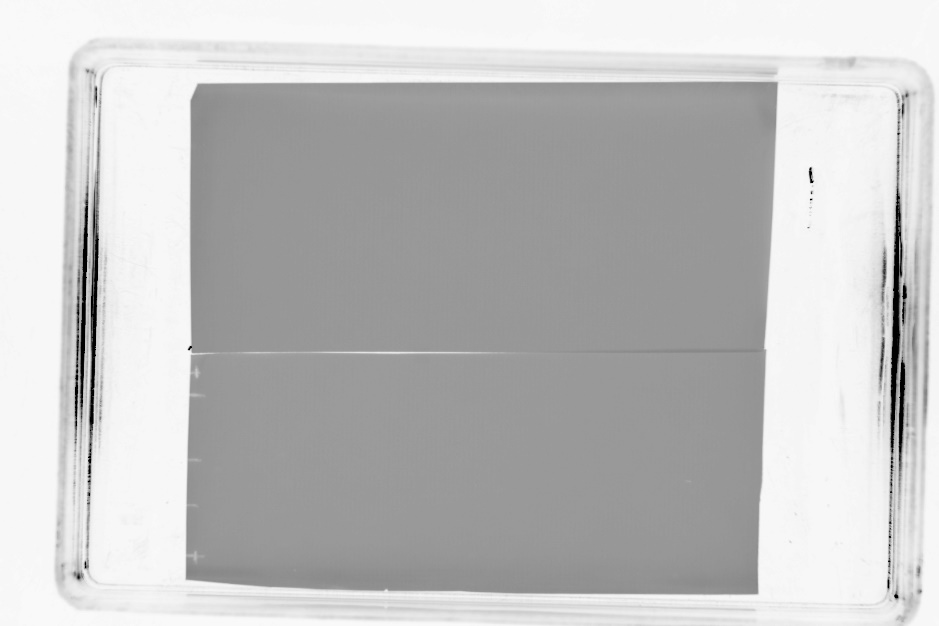**  **50**  **37**  **25**  **10**  **15** | **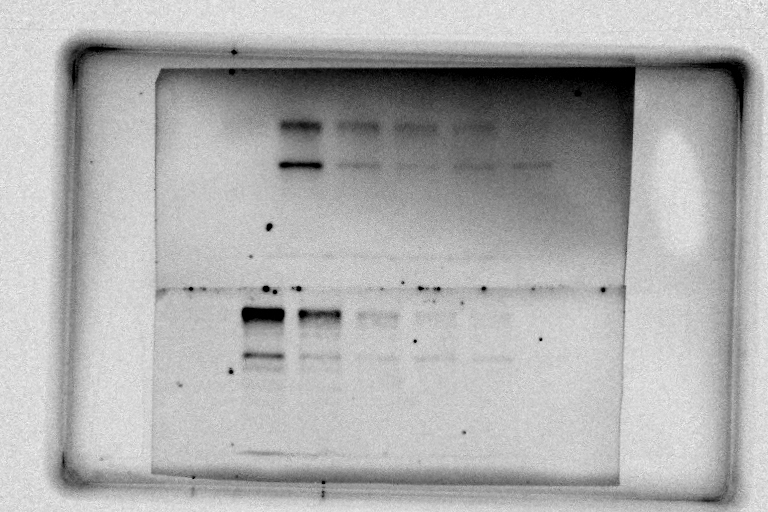**  **Pro-caspase-1**  **50**  **37**  **25**  **10**  **Intermediate**  **Caspase-1**  **15** |
| **Exp.3** | **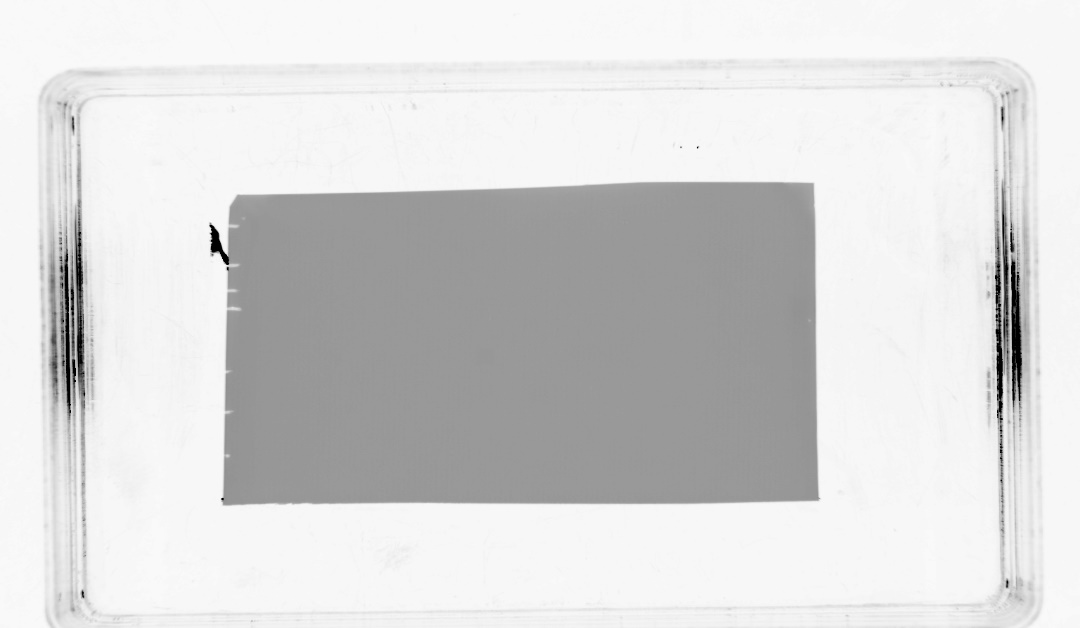**  **50**  **37**  **25**  **10**  **100**  **15**  **75** | **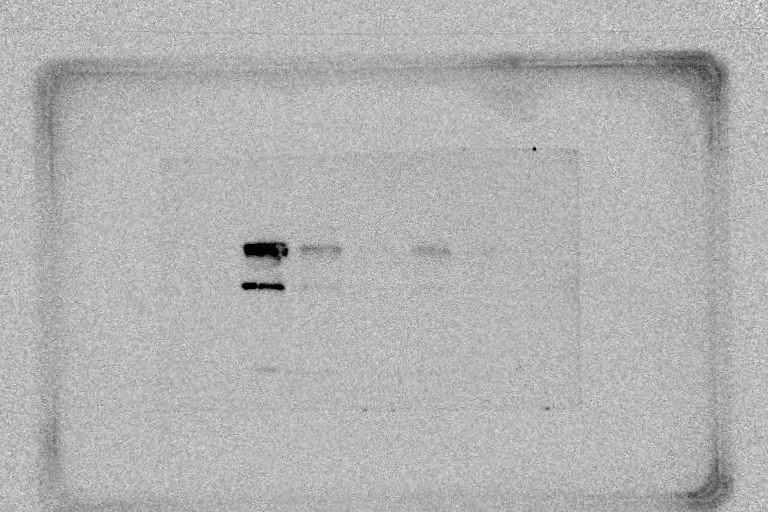**  **Caspase-1**  **Intermediate**  **Pro-caspase-1**  **50**  **37**  **25**  **10**  **100**  **75**  **15** |

| **IL-1β** | **A: Exposure time 1/100 sec** | **B: Exposure time 600 sec** |
| --- | --- | --- |
| **Exp.1** | 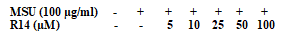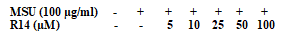 **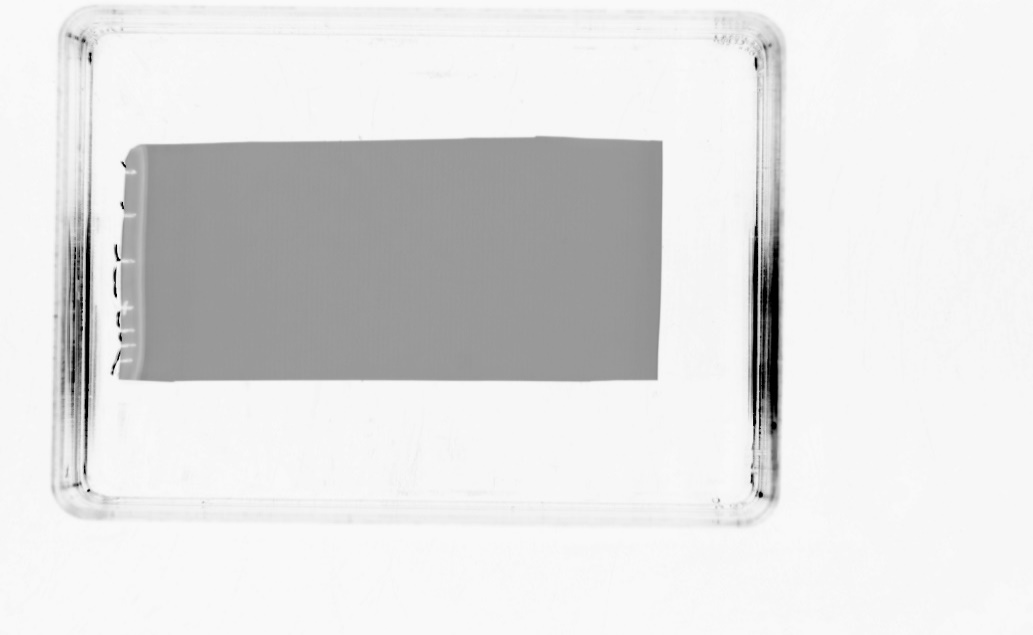**  **17**  **20**  **25**  **35**  **48**  **11** | 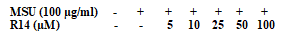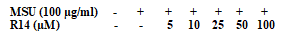 **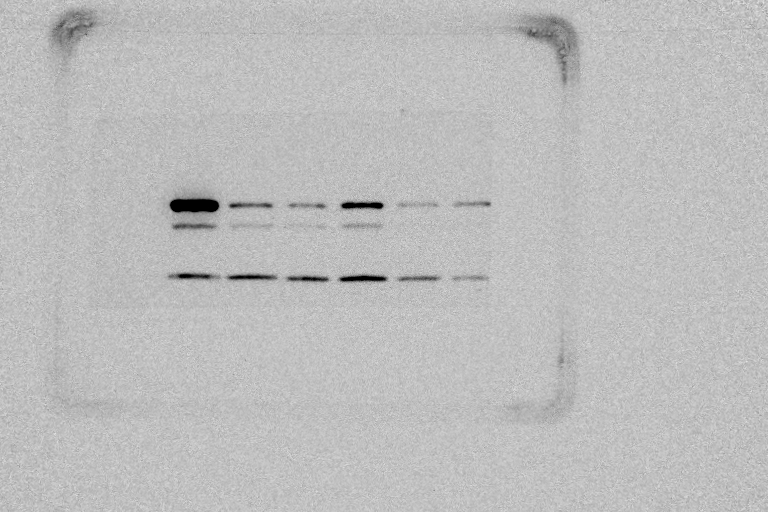**  **11**  **17**  **20**  **25**  **35**  **48**  **IL-1β**  **Pro-IL-1β** |
| **Exp.2** | **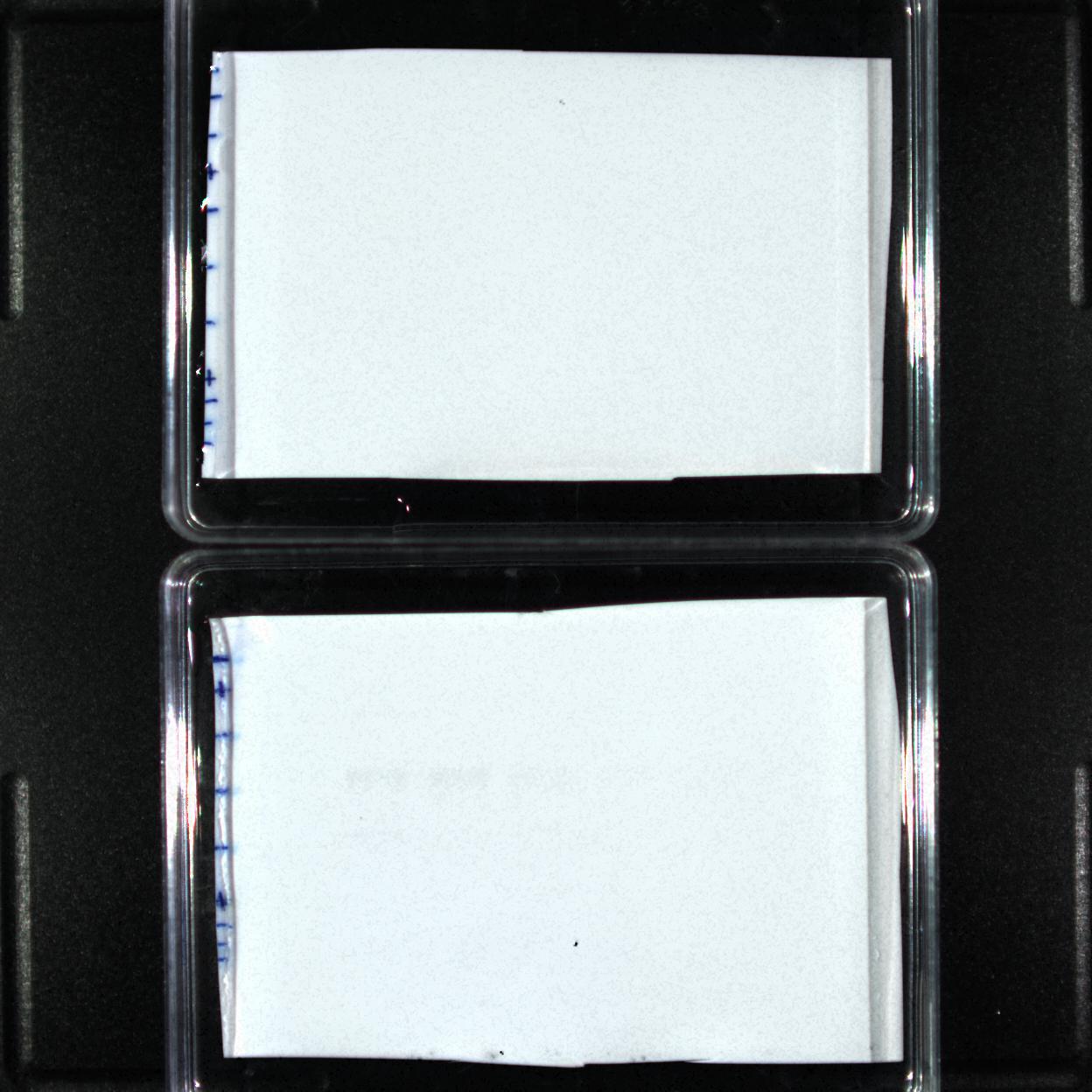**  **17**  **20**  **25**  **35**  **48** | 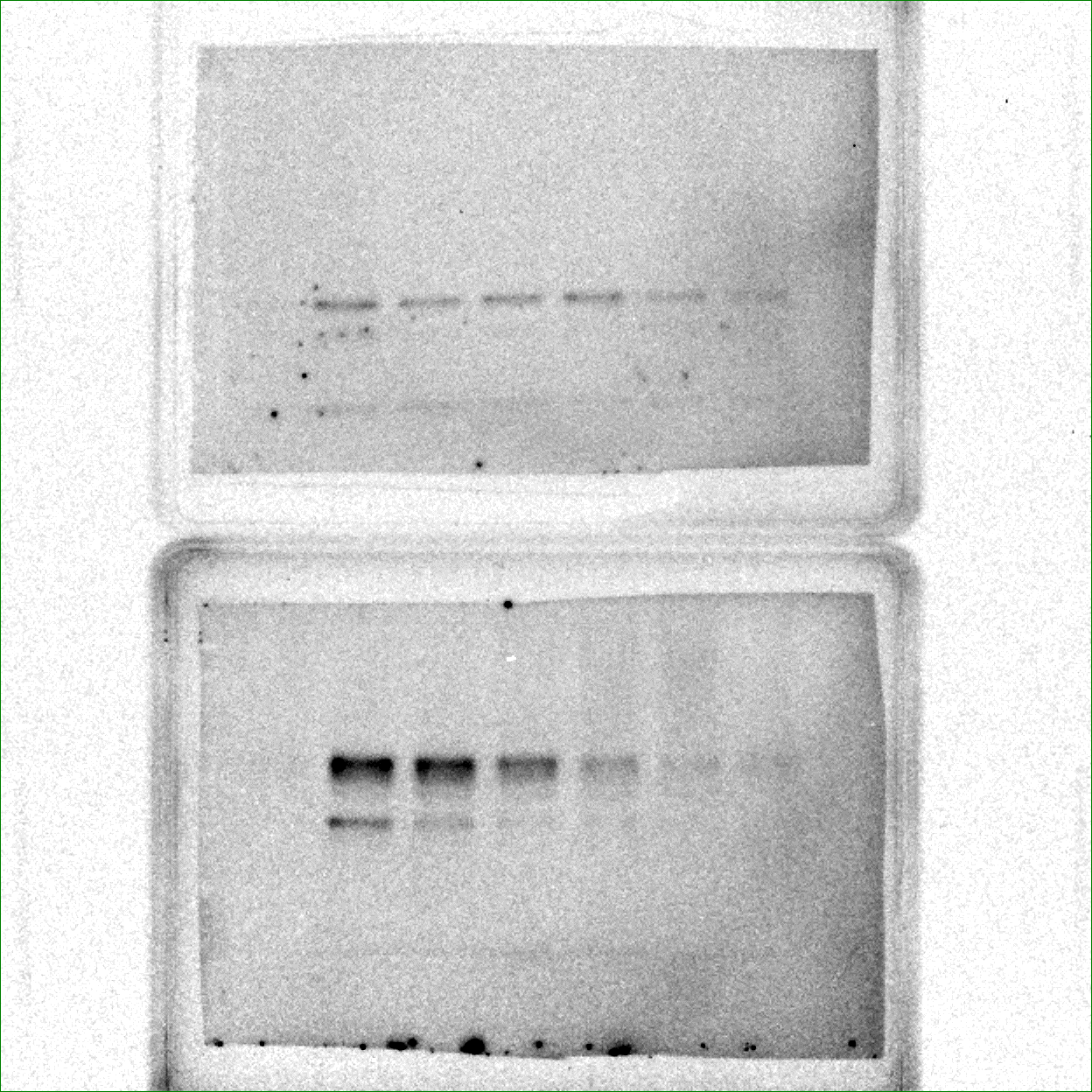  **17**  **20**  **25**  **35**  **48**  **IL-1β**  **Pro-IL-1β** |
| **Exp.3** | 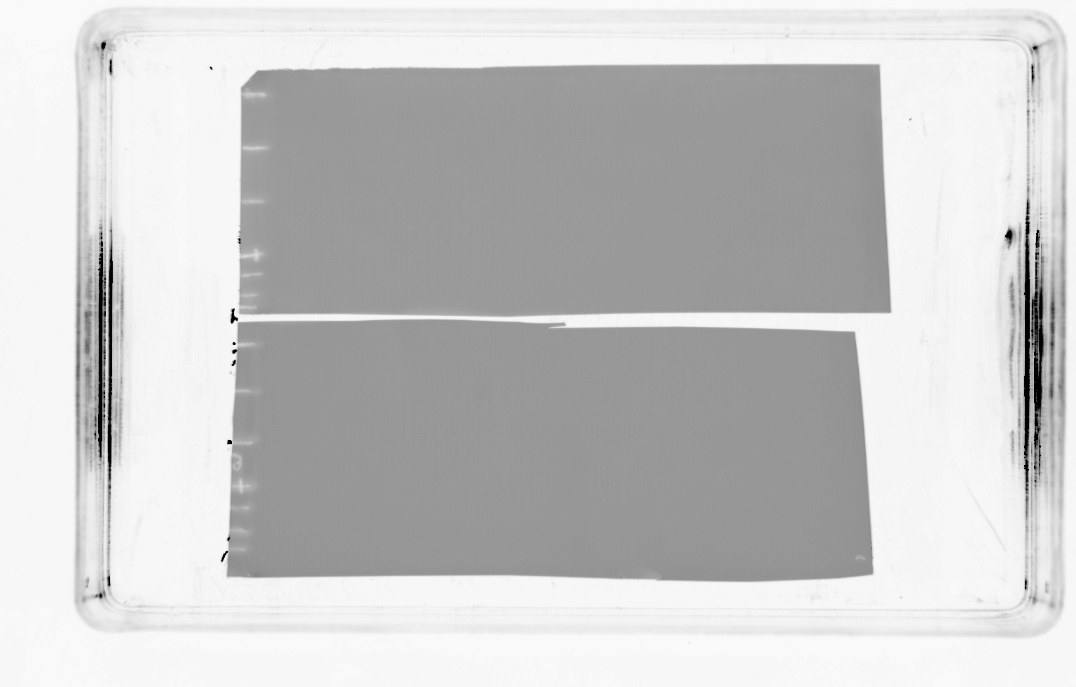  **17**  **20**  **25**  **35**  **48**  **11**  **11** | **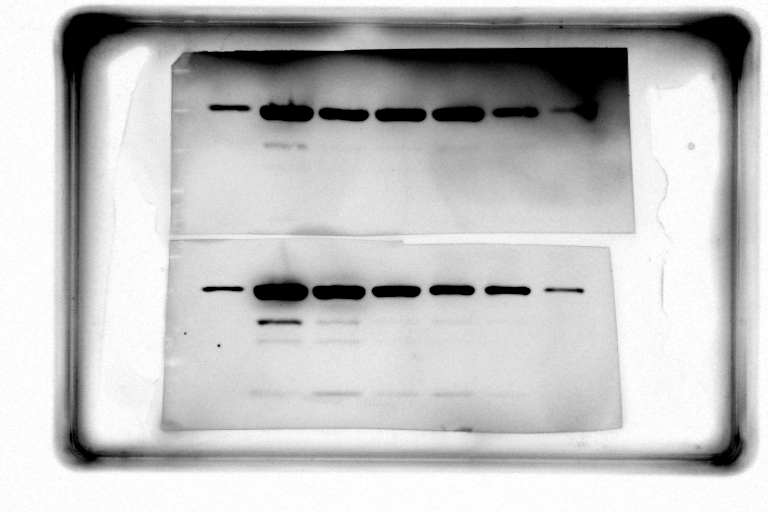**  **IL-1β**  **Pro-IL-1β**  **17**  **20**  **25**  **35**  **48**  **11**  **11** |

| **β-actin** | **A: Exposure time 1/100 sec** | **B: Exposure time 20 sec** |
| --- | --- | --- |
| **Exp.1** | **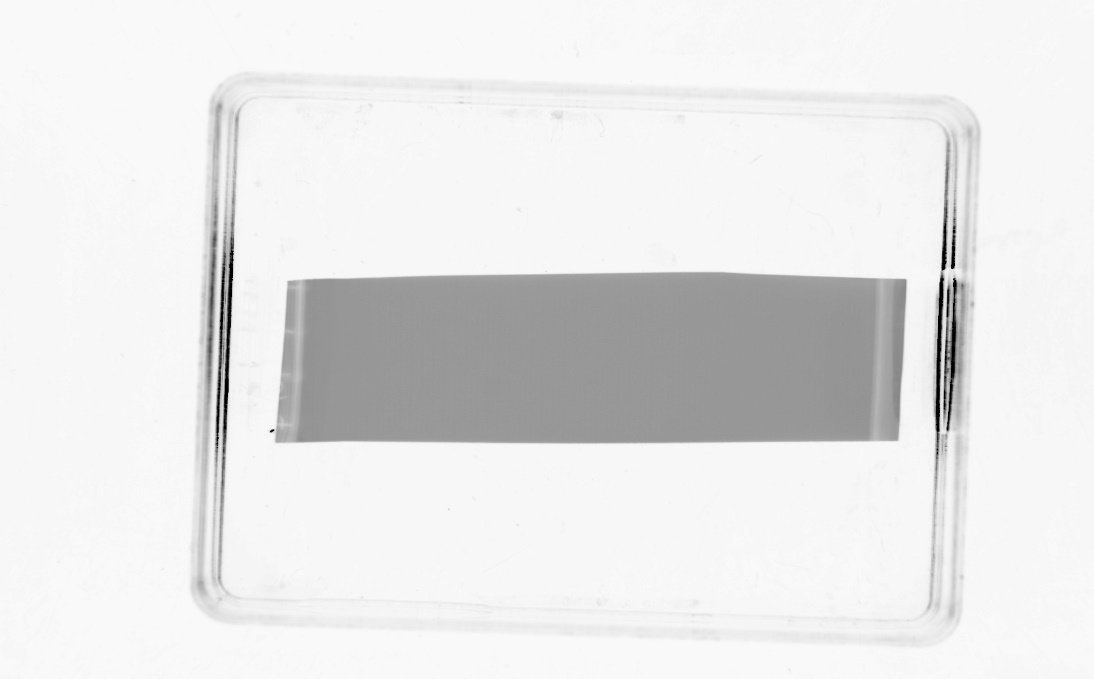** 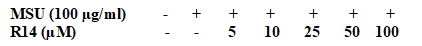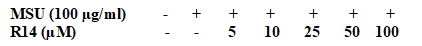 **63**  **48**  **35**  **25** | **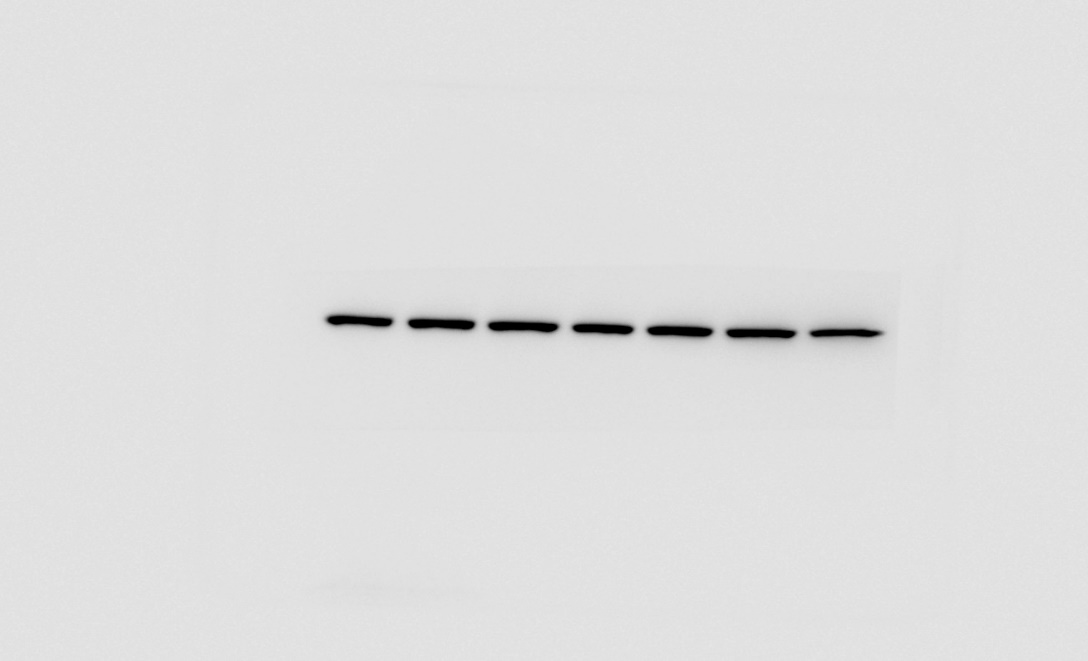**  **β-actin**  **63**  **48**  **35**  **25** 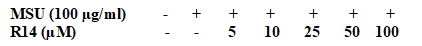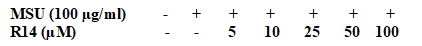 |
| **Exp.2** | 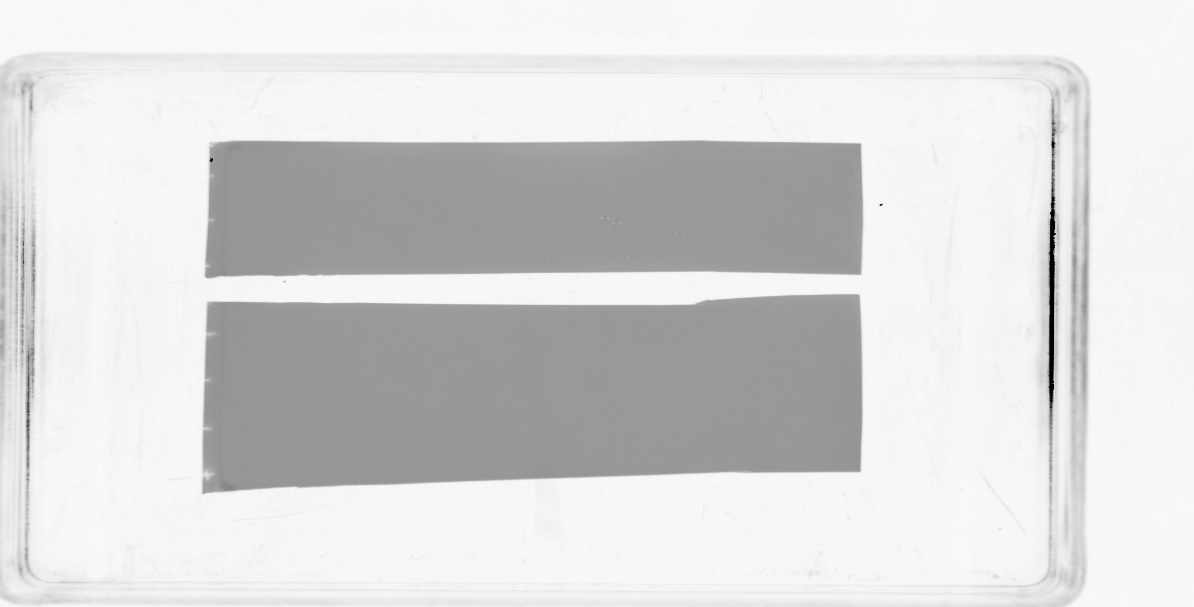  **63**  **48**  **35**  **25** | **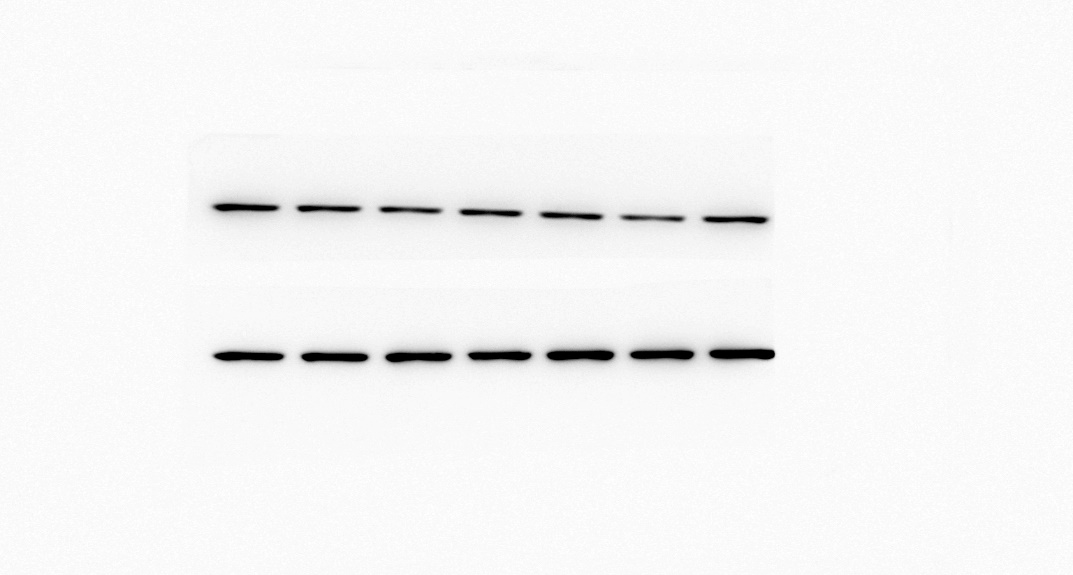**  **β-actin**  **63**  **48**  **35**  **25** |
| **Exp.3** | **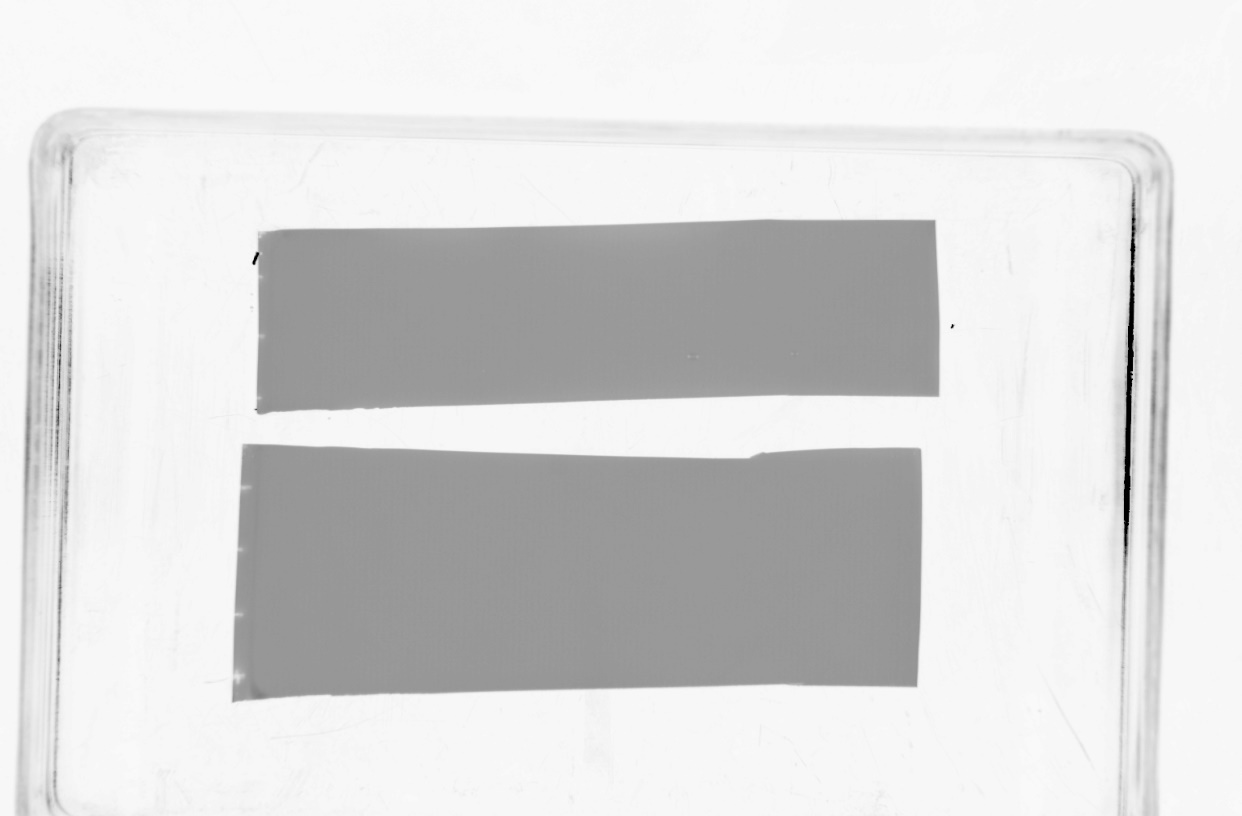**  **63**  **48**  **35**  **25** | **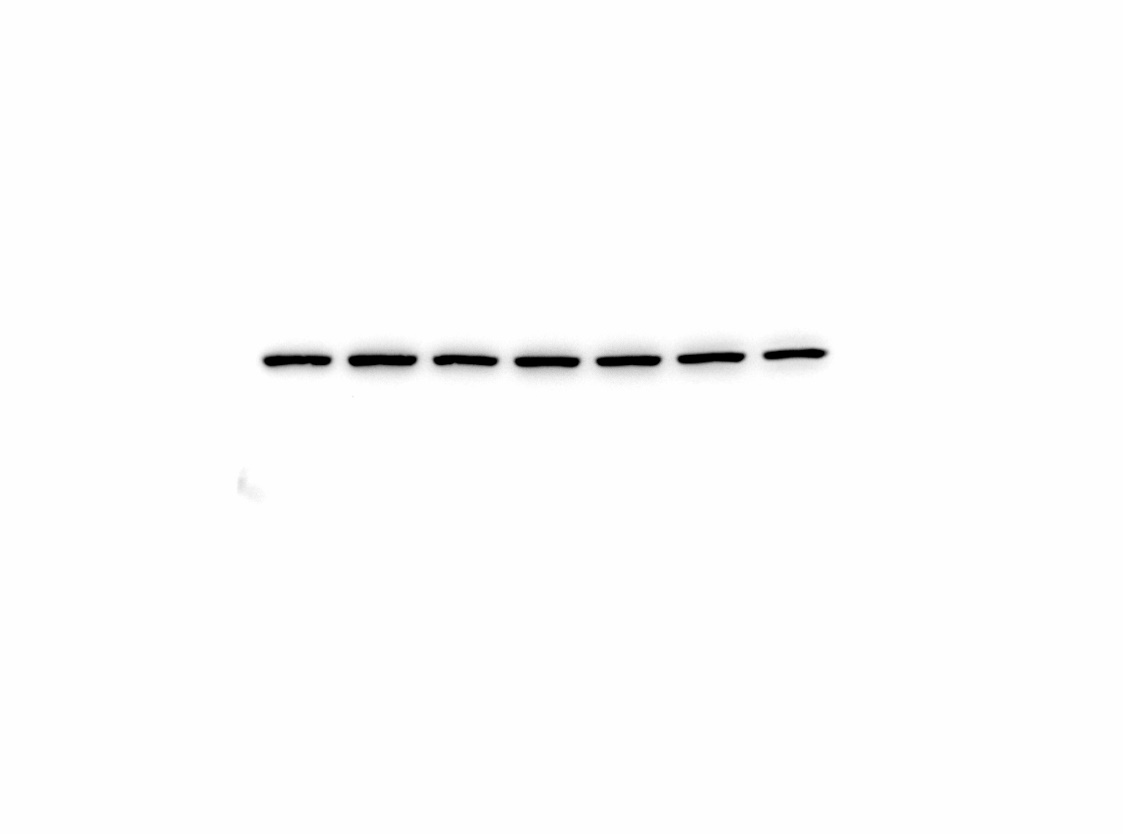**  **63**  **48**  **35**  **25**  **β-actin** |

**Proteins from cell lysates**

| **NLRP3** | **A: Exposure time 1/100 sec** | **B: Exposure time 100 sec** |
| --- | --- | --- |
| **Exp.1** | **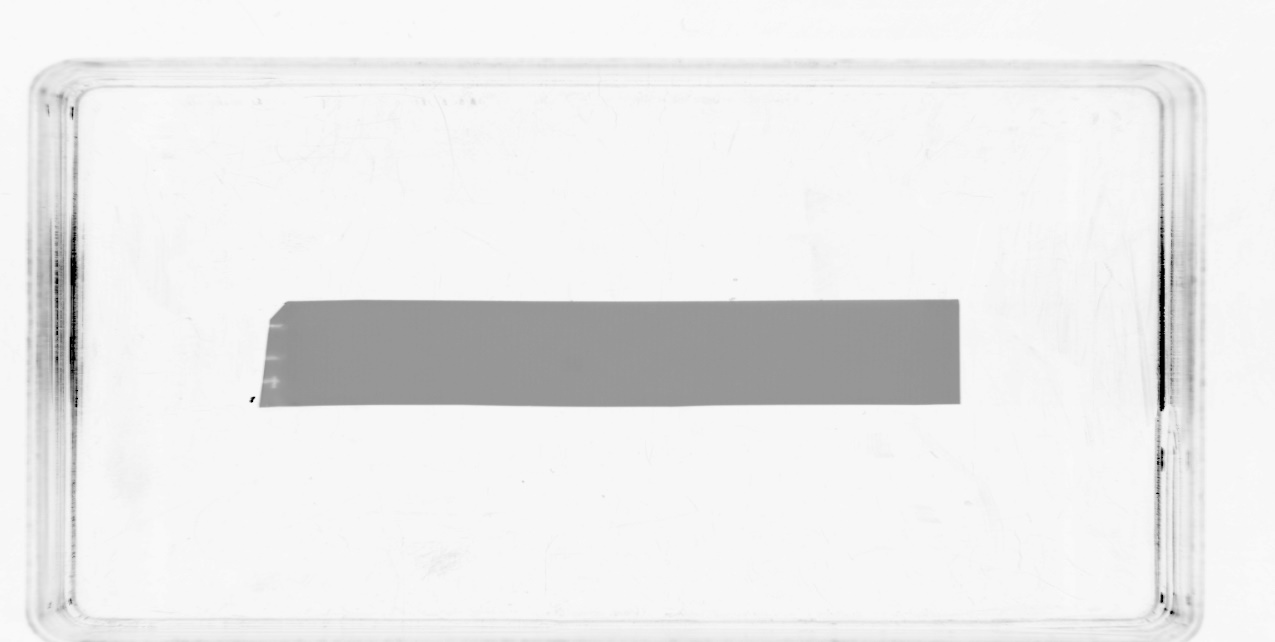** 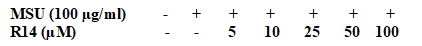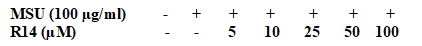 **135**  **100**  **75** 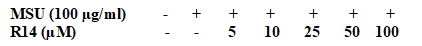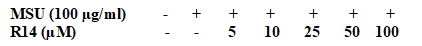 | 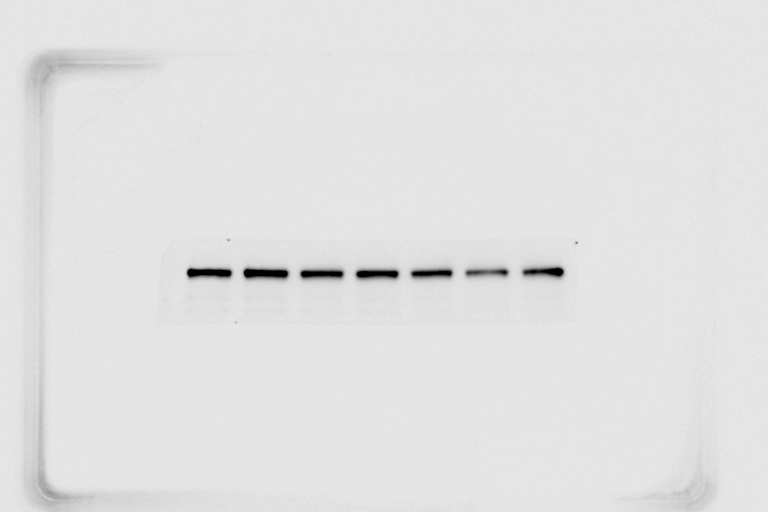  **135**  **100**  **75**  **NLRP3** |
| **Exp.2** | **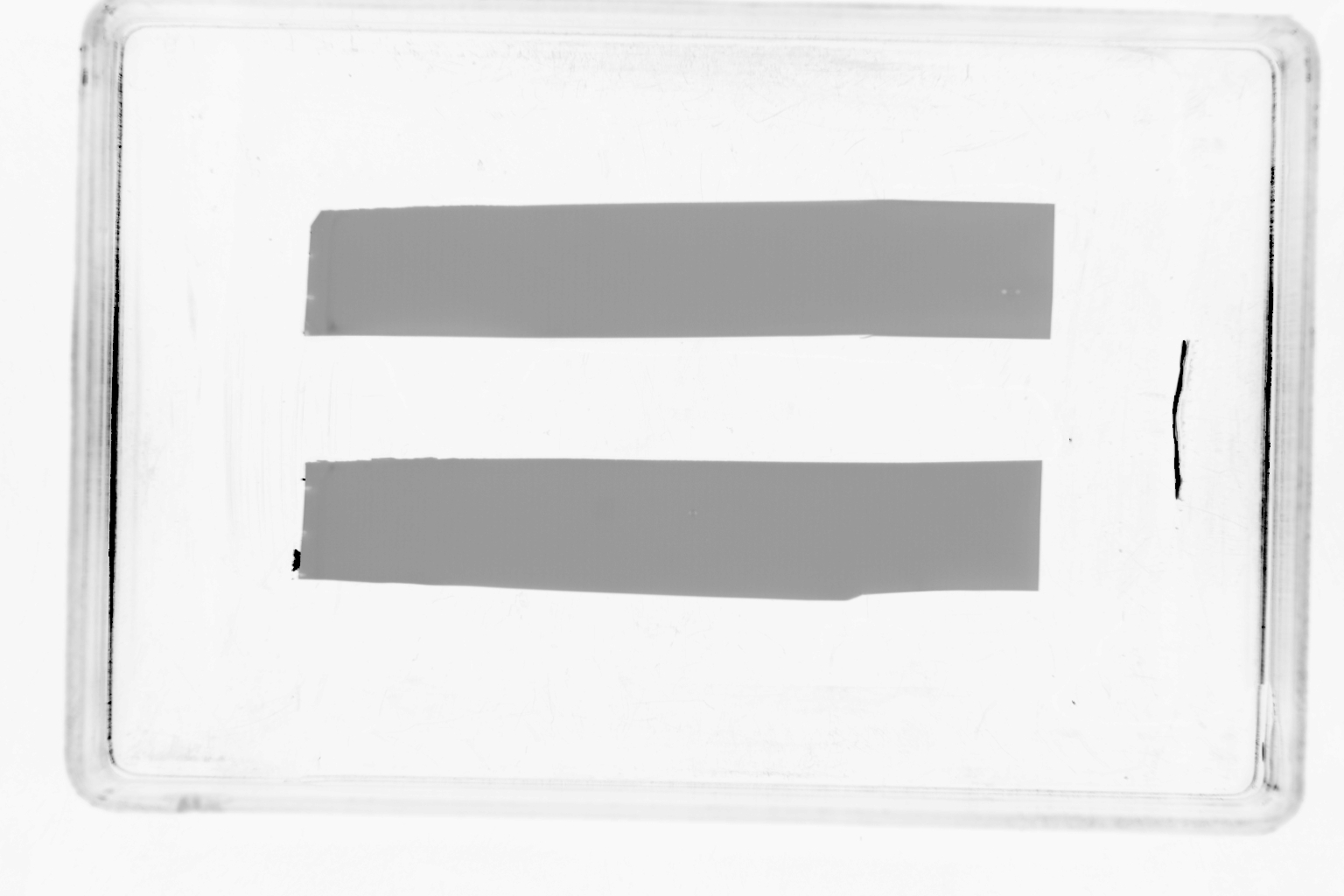**  **135**  **100**  **75** | 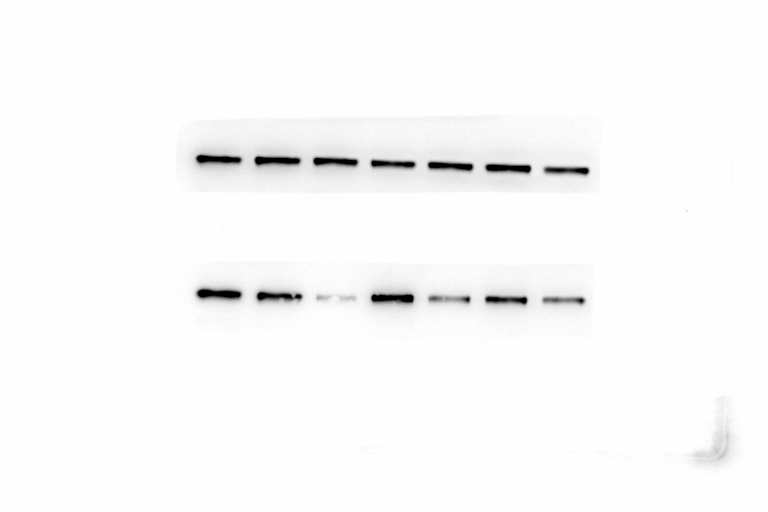  **NLRP3**  **135**  **100**  **75** |
| **Exp.3** | **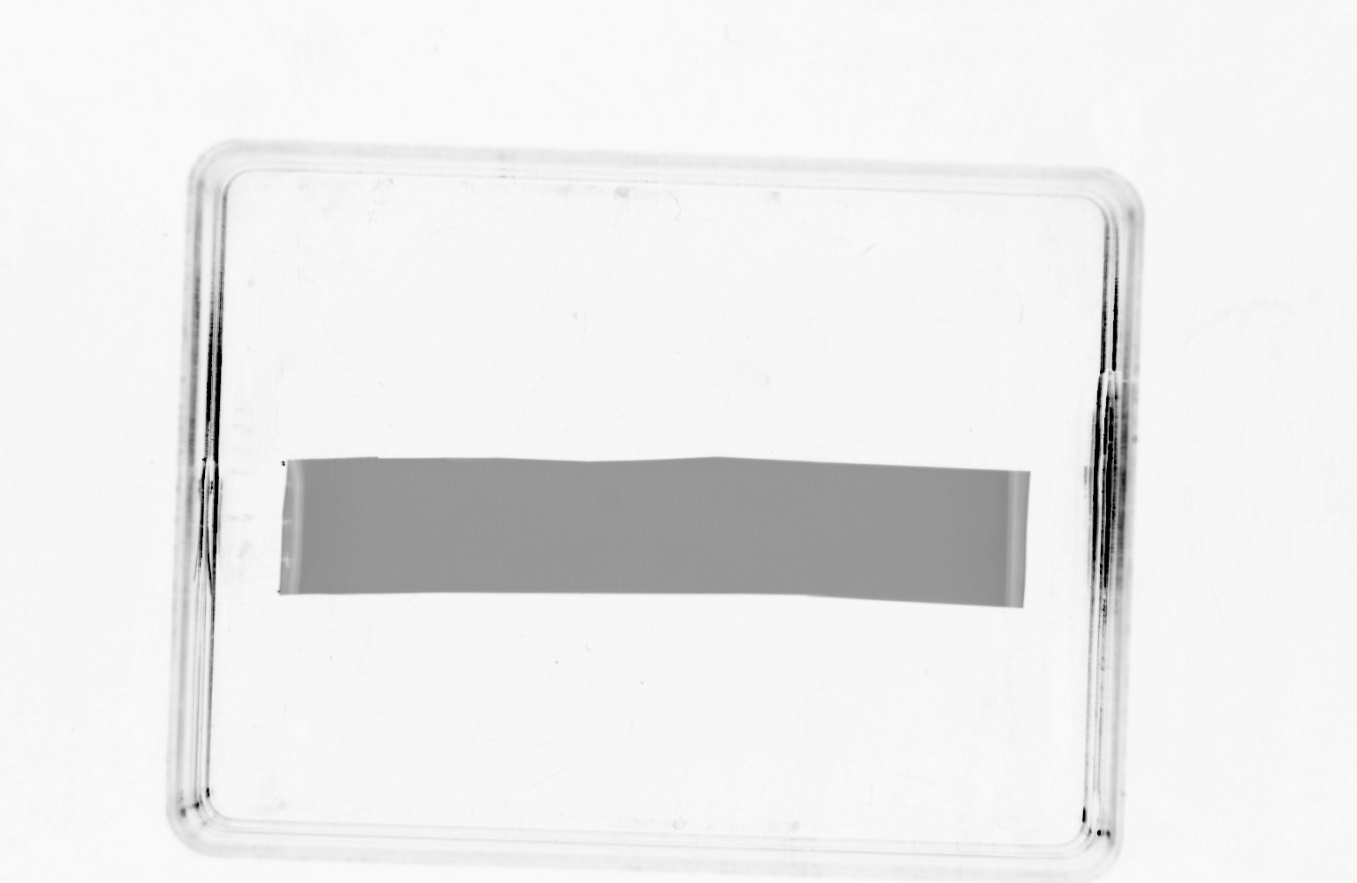**  **135**  **100**  **75** | 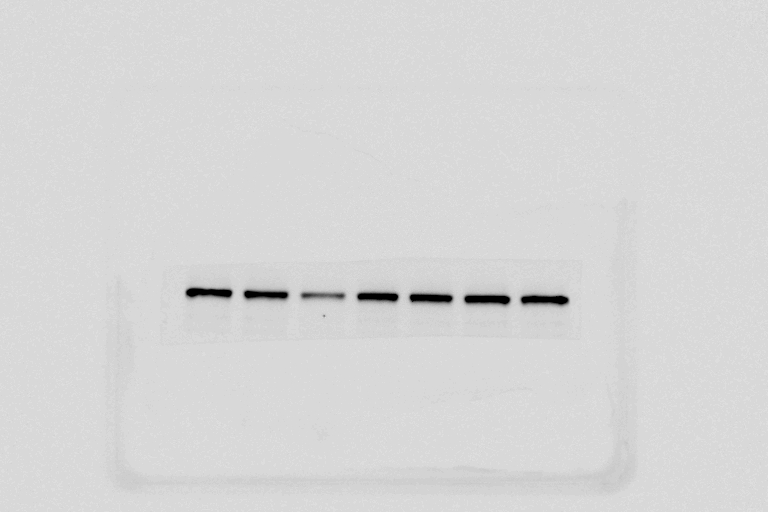  **NLRP3**  **135**  **100**  **75** |

| **β-actin** | **A: Exposure time 1/100 sec** | **B: Exposure time 20 sec** |
| --- | --- | --- |
| **Exp.1** | **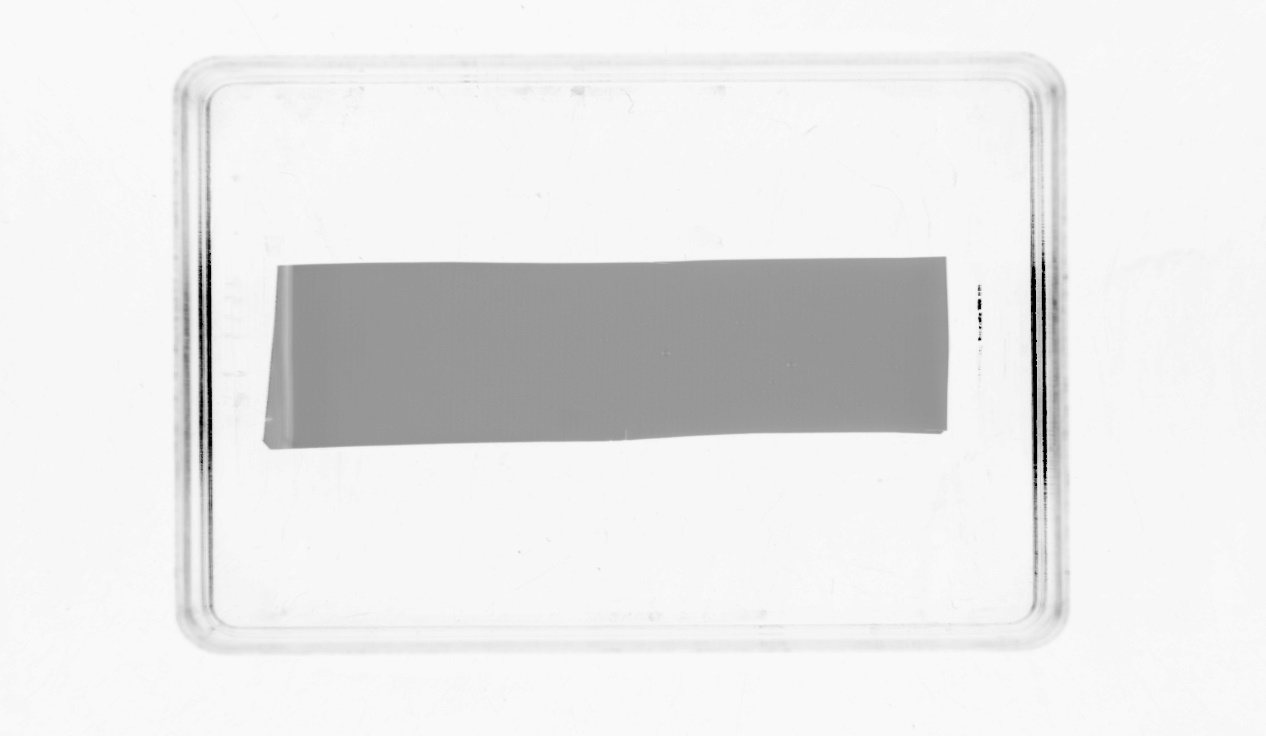** 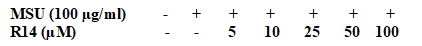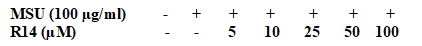 **63**  **48**  **35**  **25** | 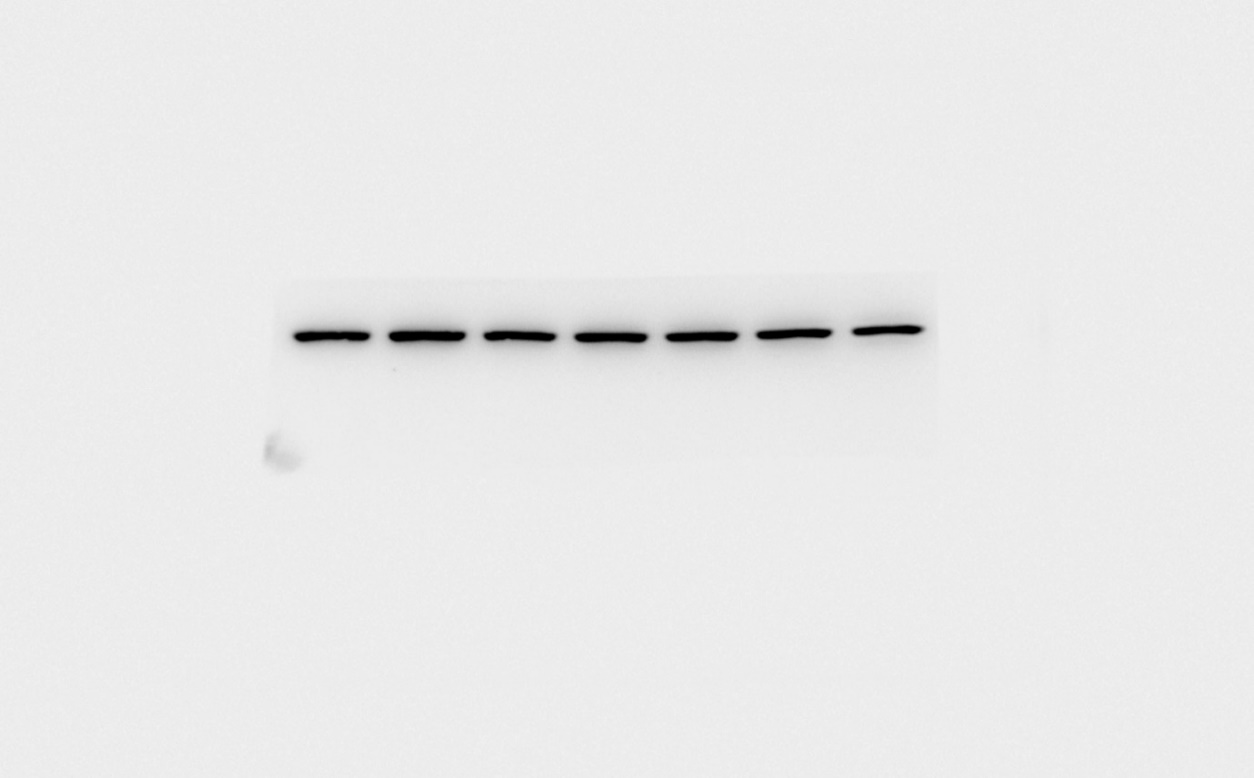  **63**  **48**  **35**  **25** 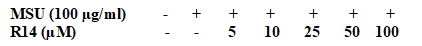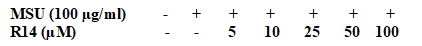 **β-actin** |
| **Exp.2** | 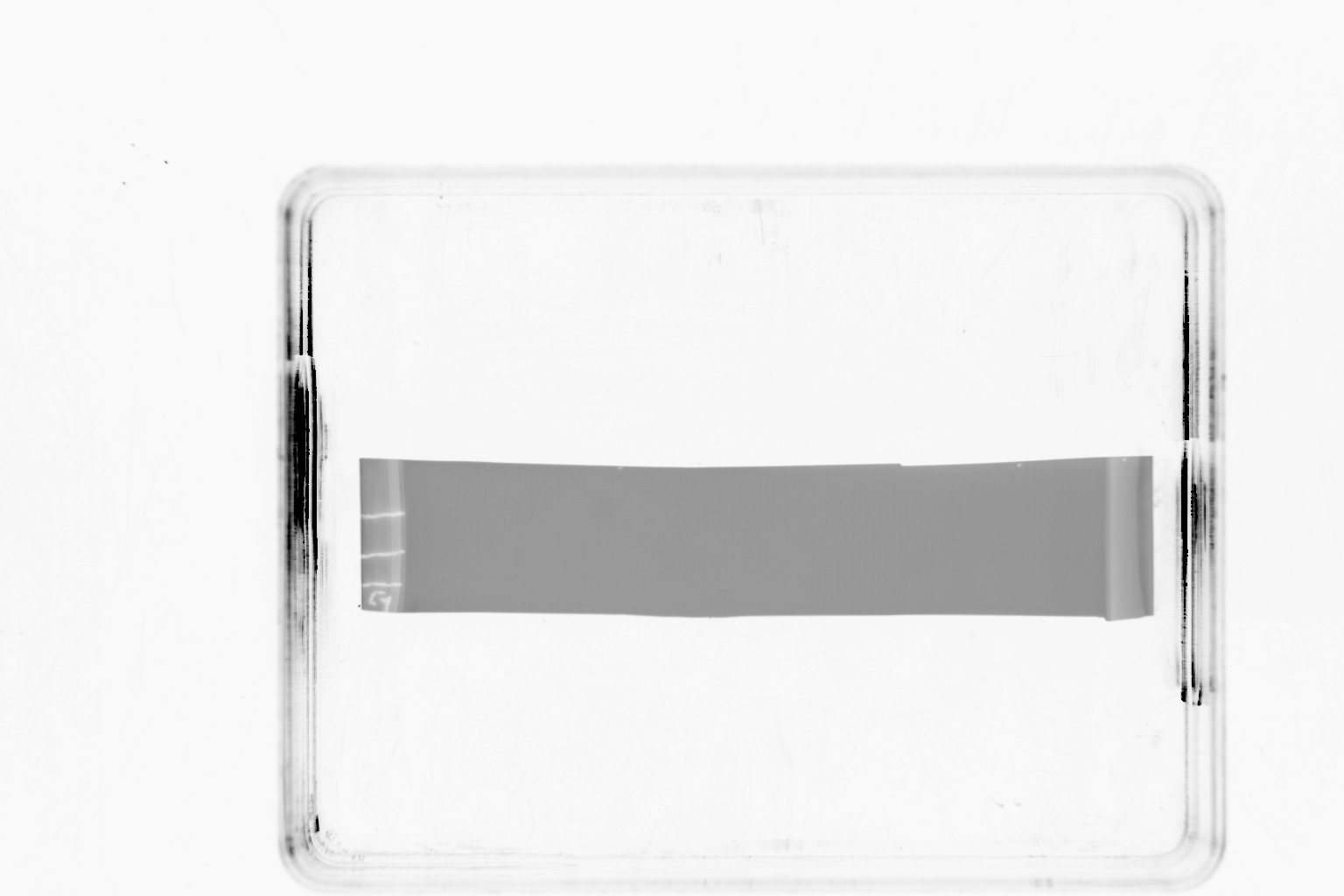  **48**  **35**  **63**  **25** | 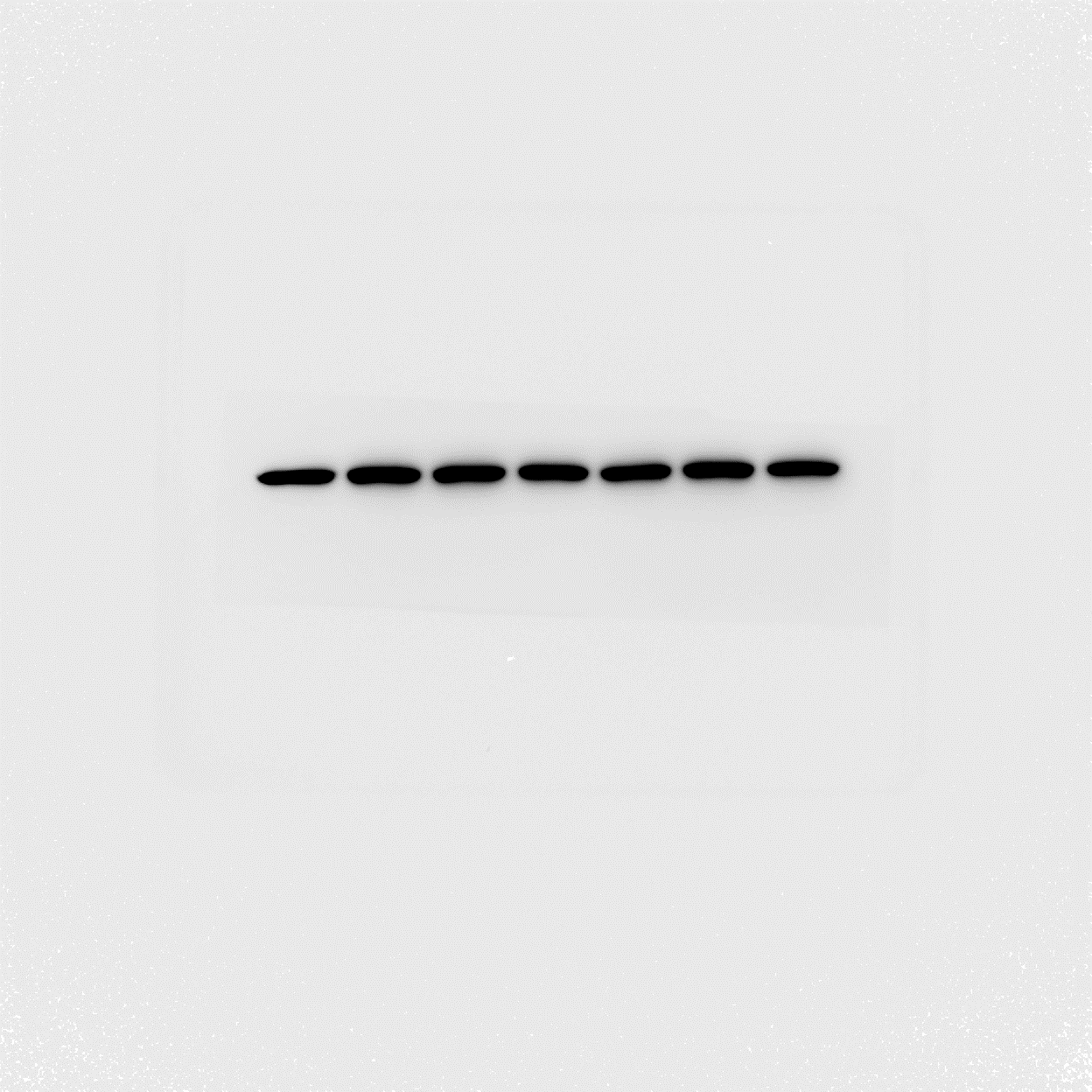  **48**  **35**  **63**  **25**  **β-actin** |
| **Exp.3** | **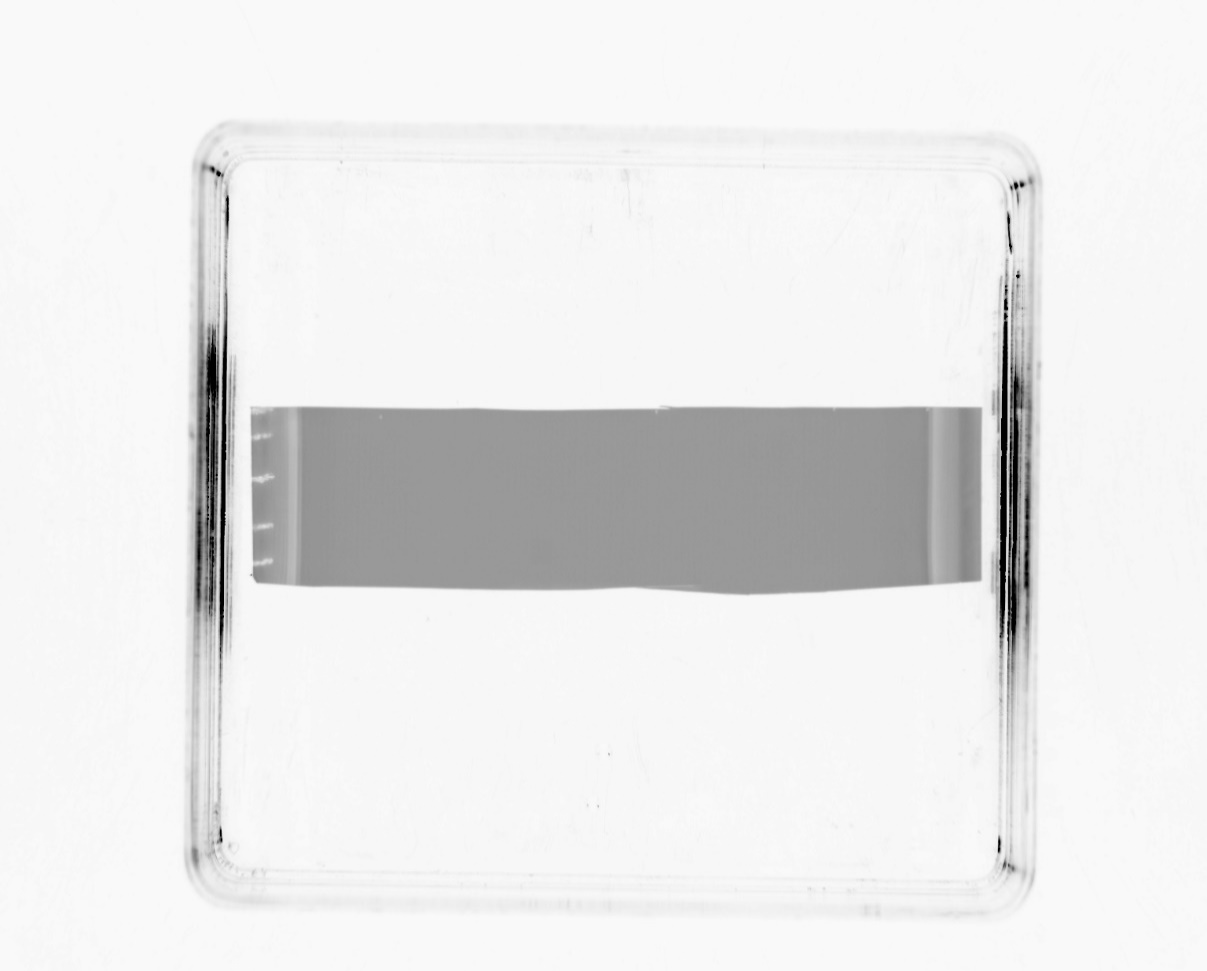**  **17**  **20**  **48**  **35**  **25** | 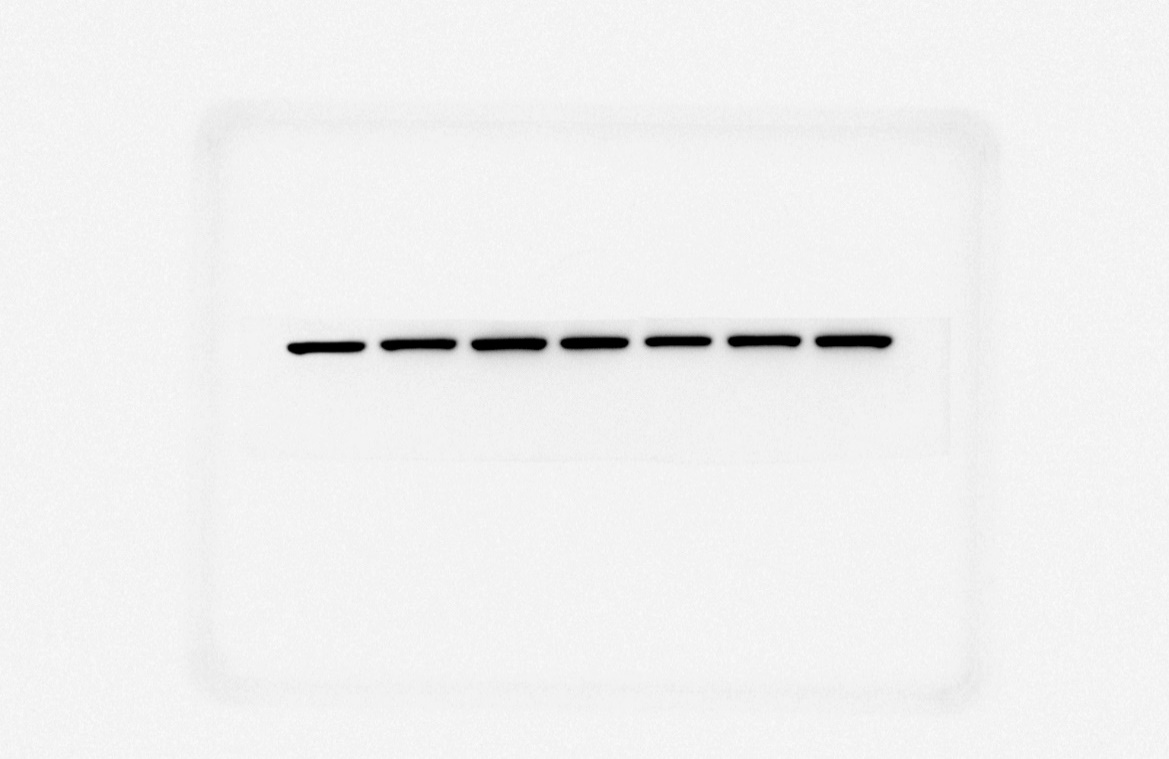  **17**  **20**  **48**  **35**  **25**  **β-actin** |

**Figure 4**

**Proteins from Nuclear extraction**

**48**

| **NF-κB p65** | **A: Exposure time 1/100 sec** | **B: Exposure time 240 sec** |
| --- | --- | --- |
| **Exp.1**  **48**  **75**  **63** | 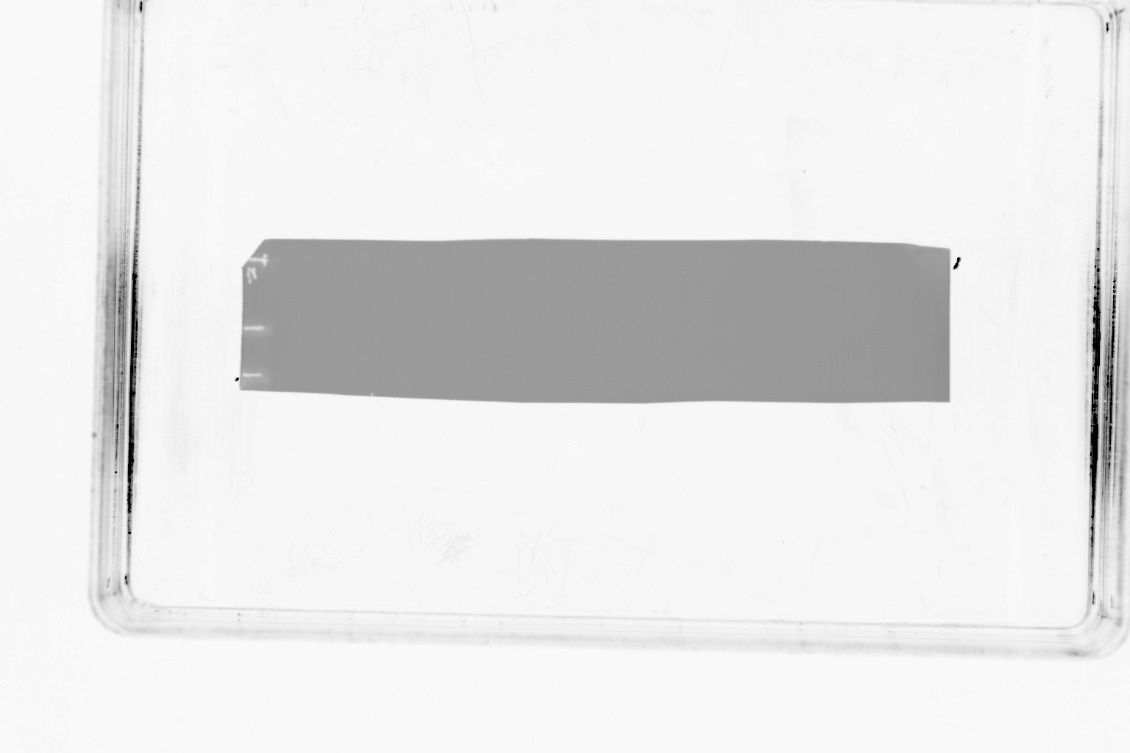 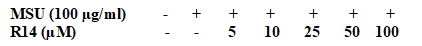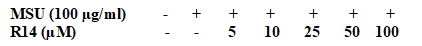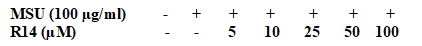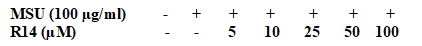 | **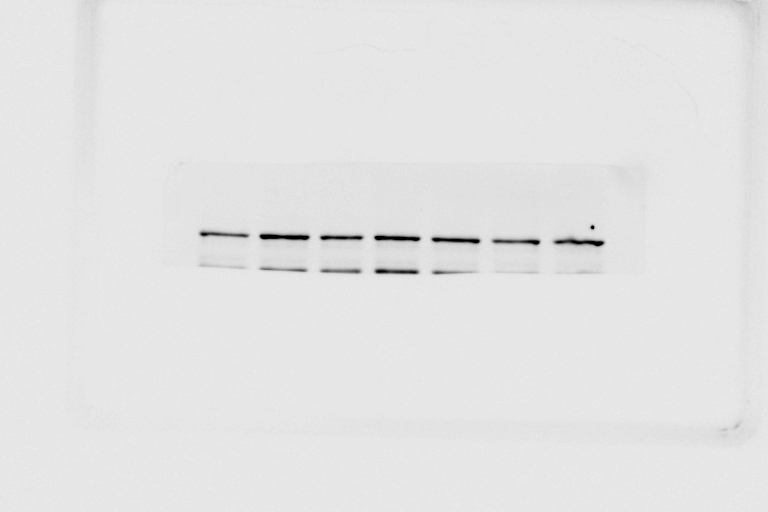**  **75**  **63**  **p65** |
| **Exp.2** | **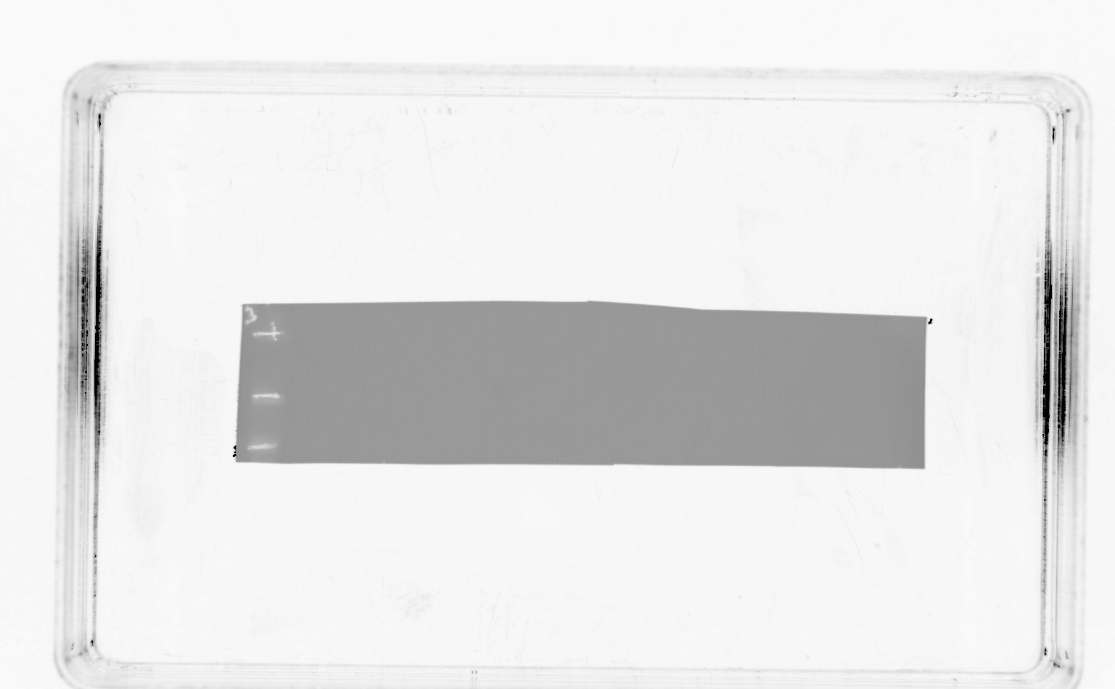**  **75**  **63** | 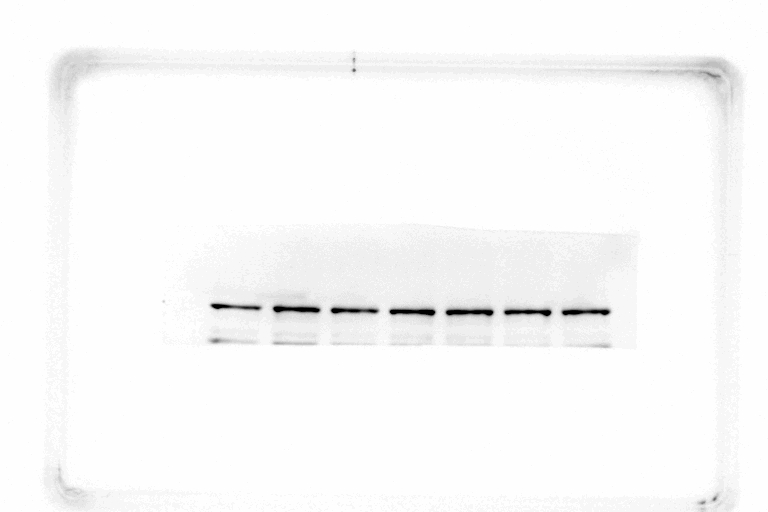  **48**  **75**  **63**  **p65** |
| **Exp.3**  **75**  **63** | **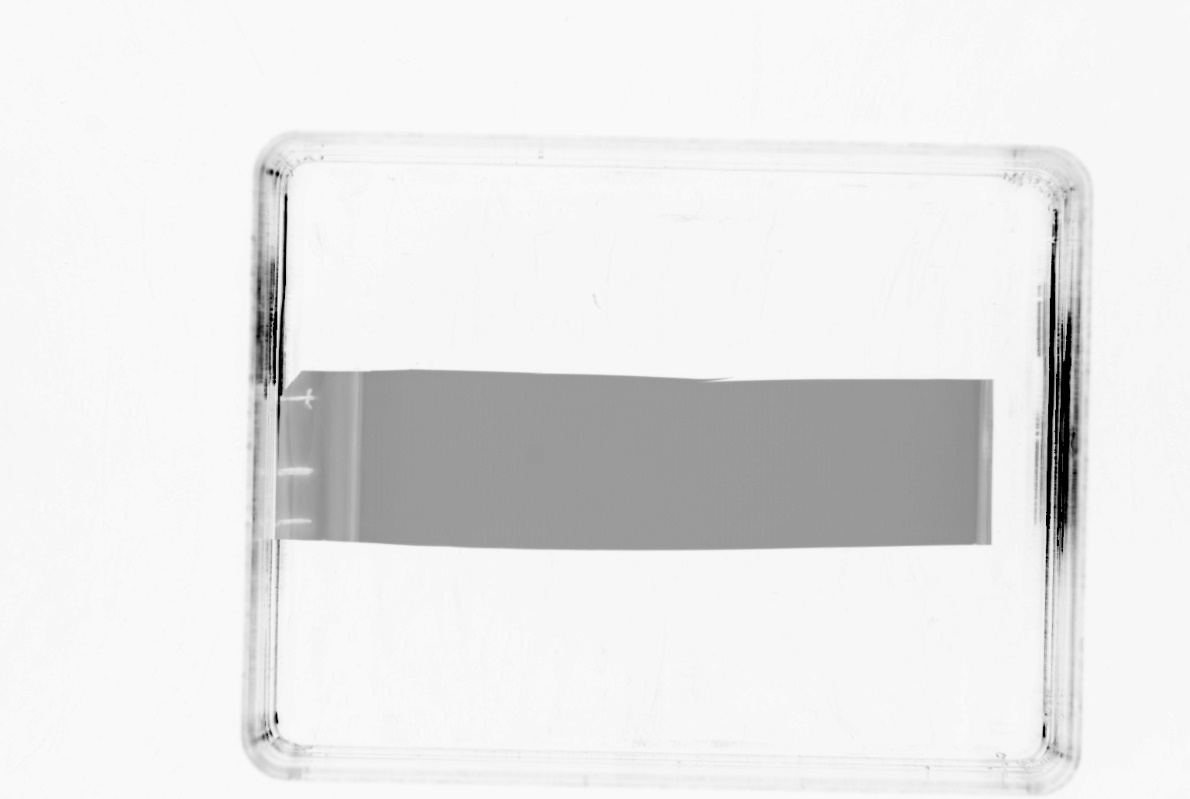**  **48** | 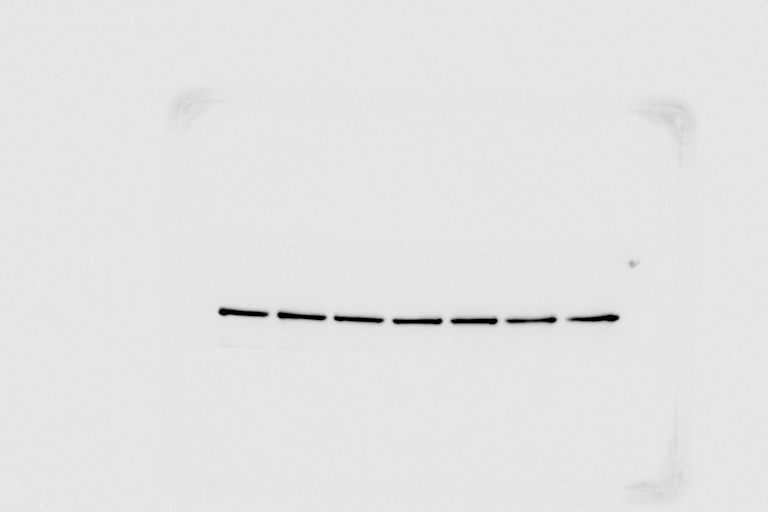  **p65**  **75**  **63**  **48**  **48**  **48** |

| **β-actin** | **A: Exposure time 1/100 sec** | **B: Exposure time 20 sec** |
| --- | --- | --- |
| **Exp.1** | **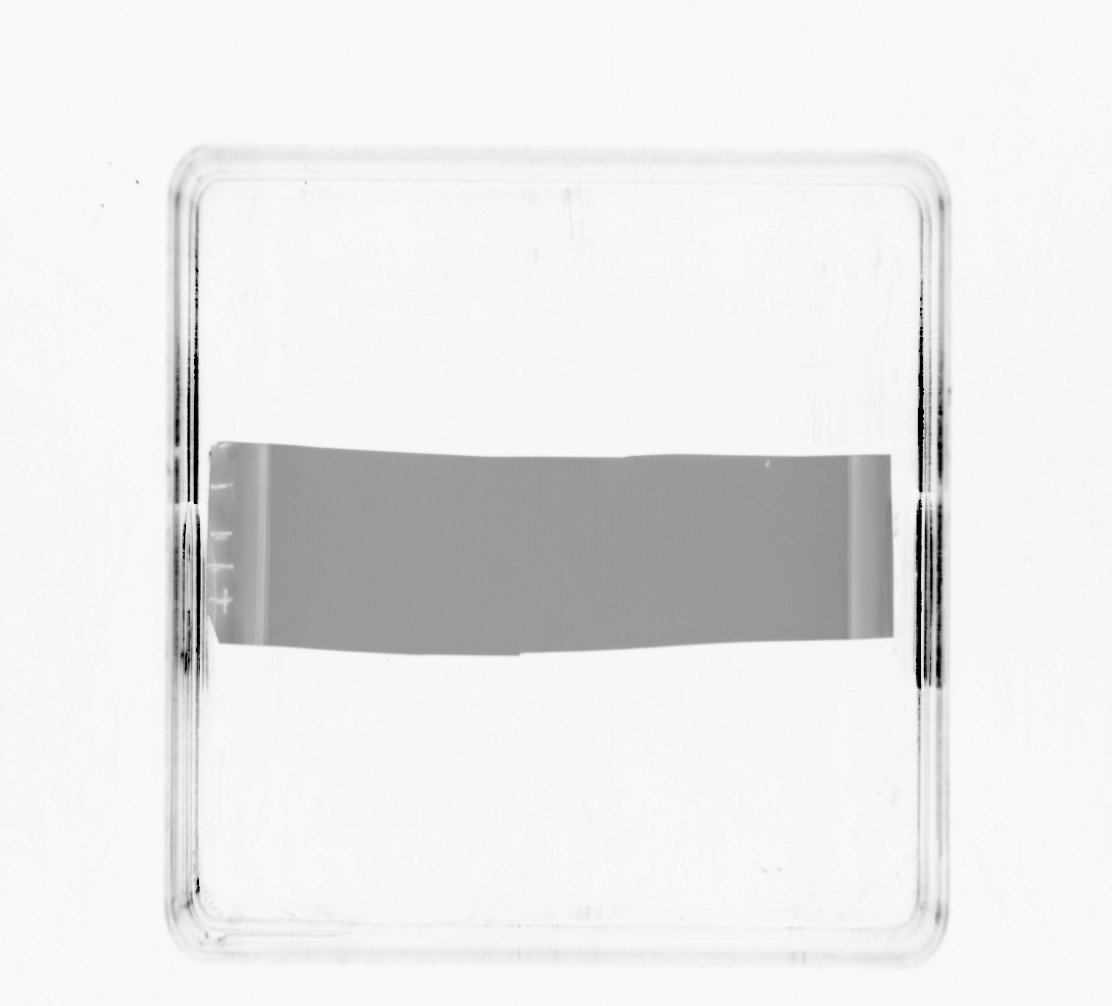** 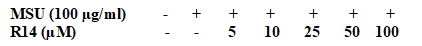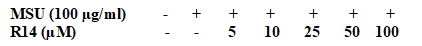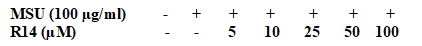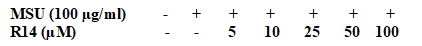 **17**  **35**  **25**  **48**  **20** | **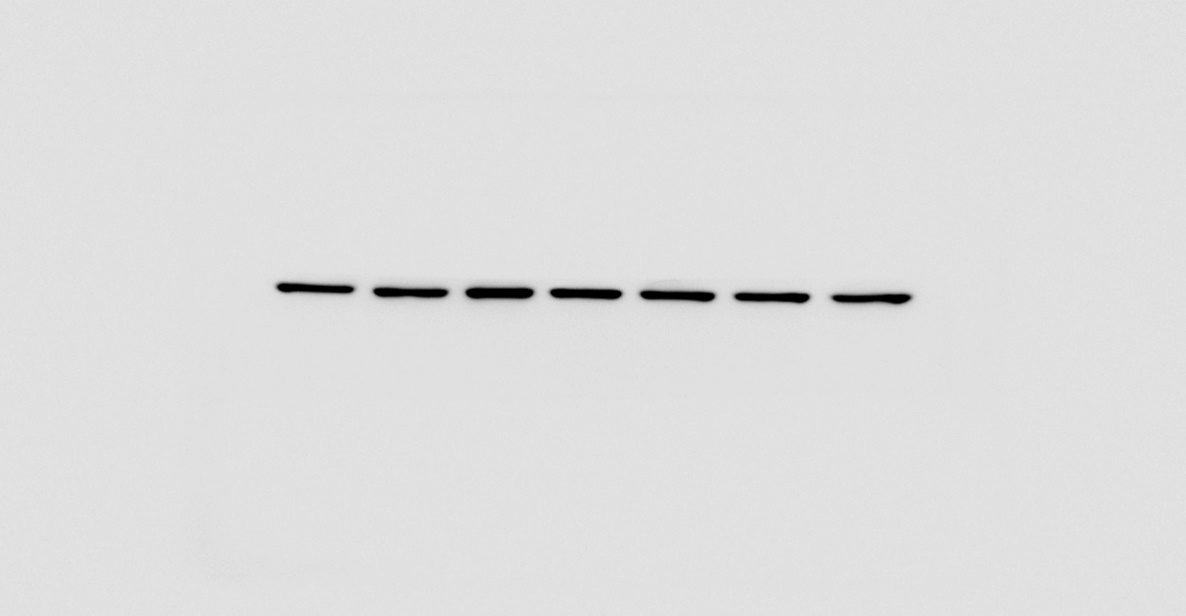**  **35**  **25**  **48**  **20**  **17**  **β-actin** |
| **Exp.2** | 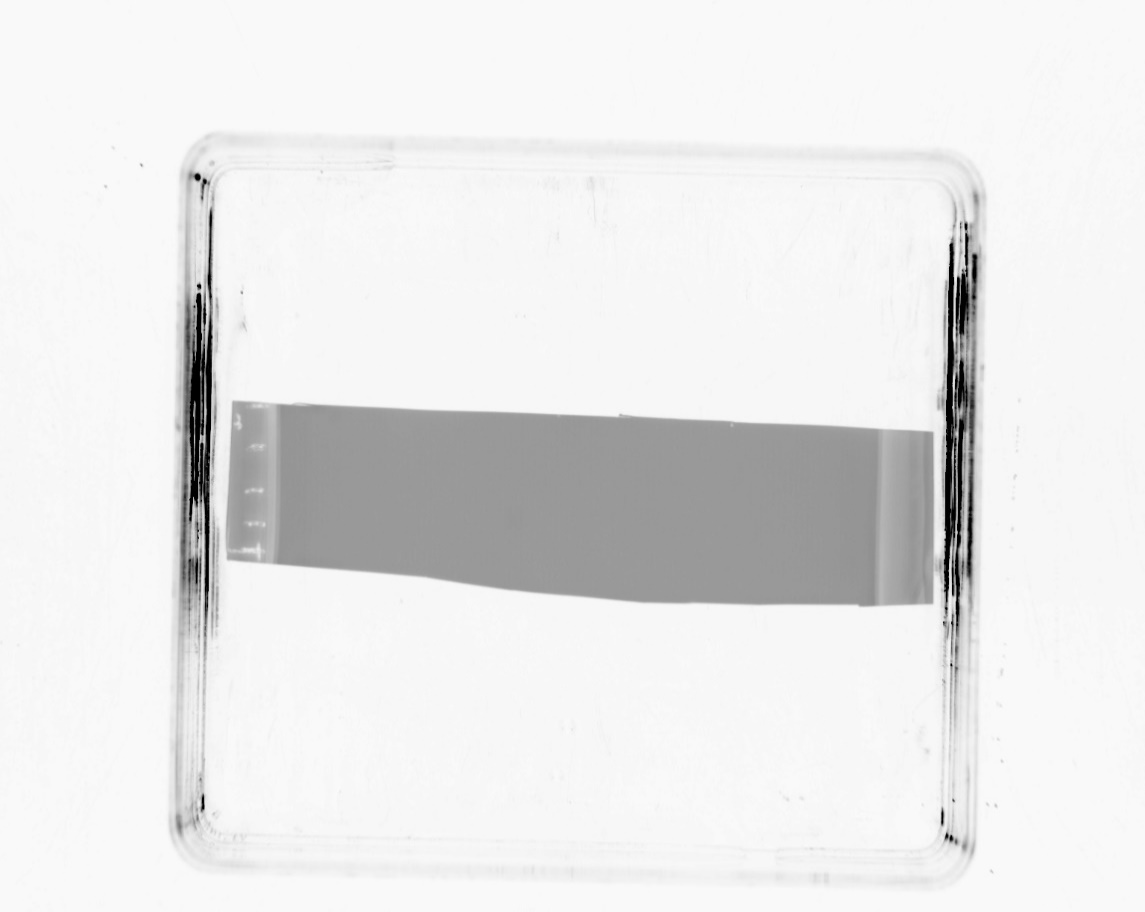  **17**  **35**  **25**  **48**  **20** | 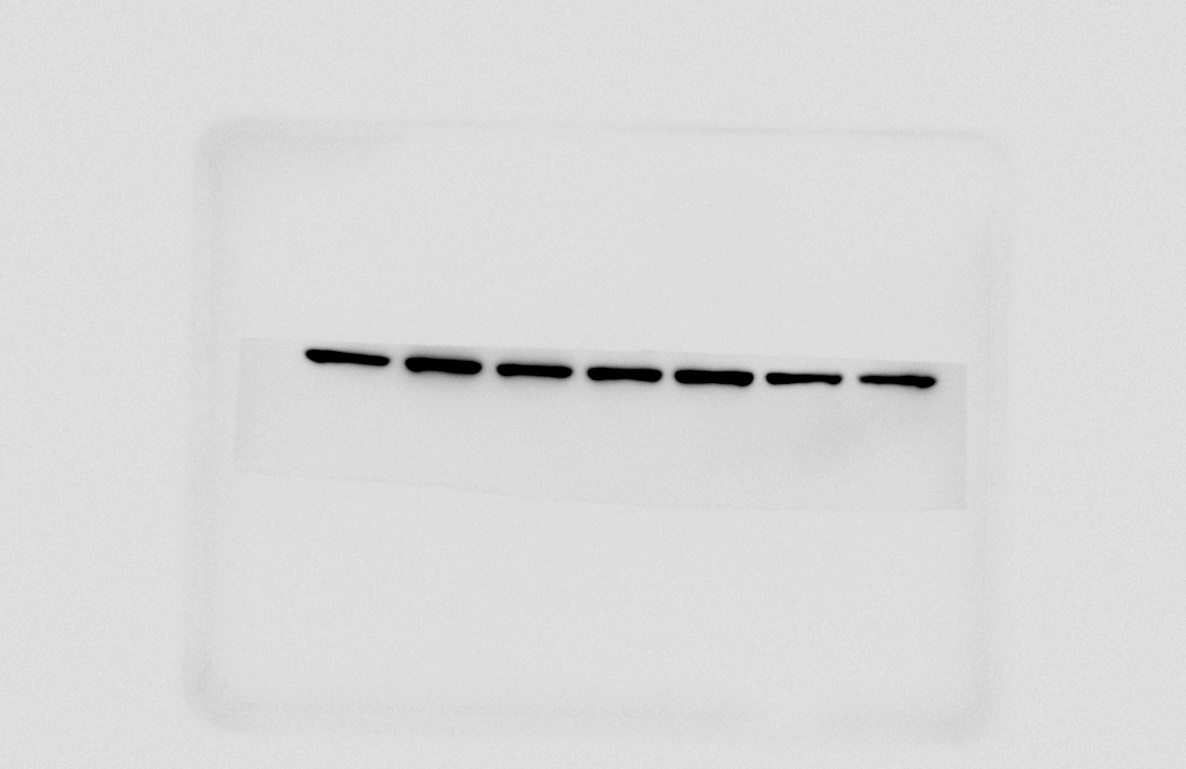  **β-actin**  **35**  **25**  **48**  **20** |
| **Exp.3** | 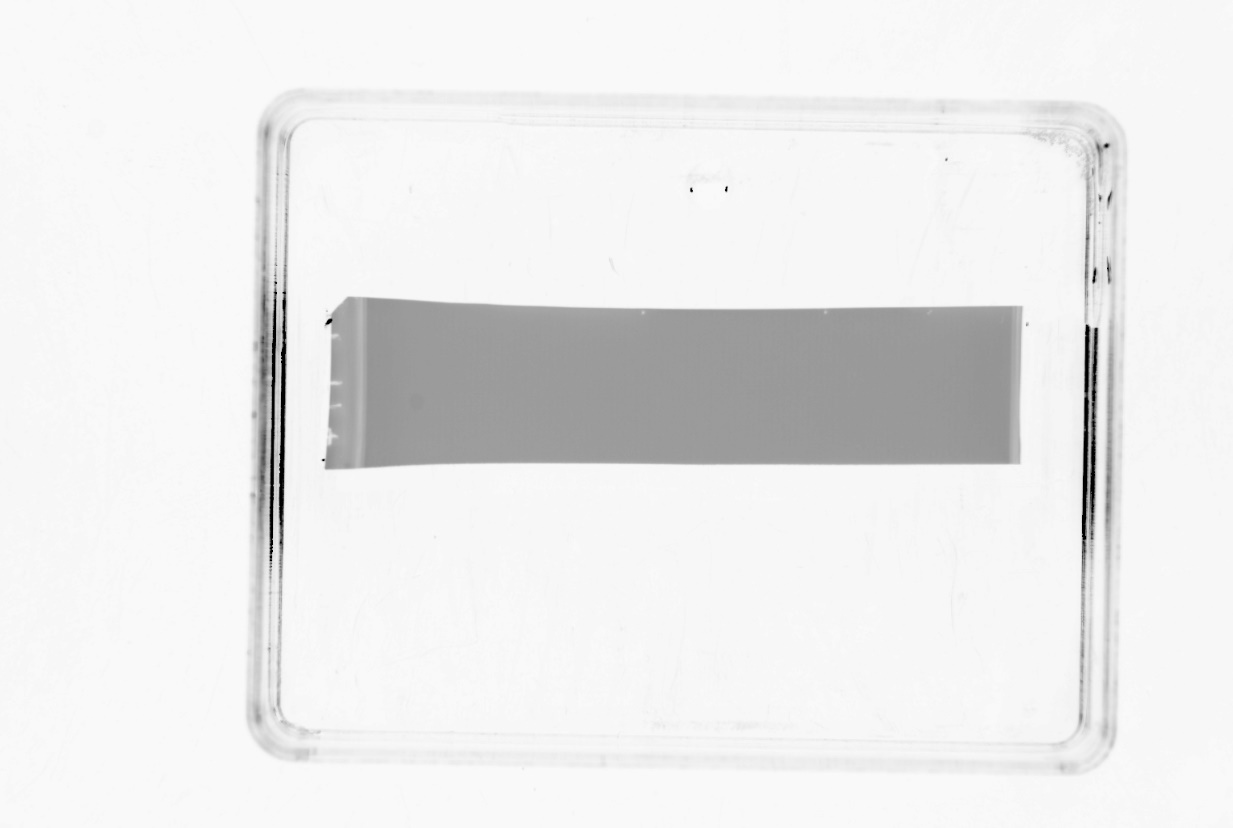  **48**  **35**  **25**  **20** | 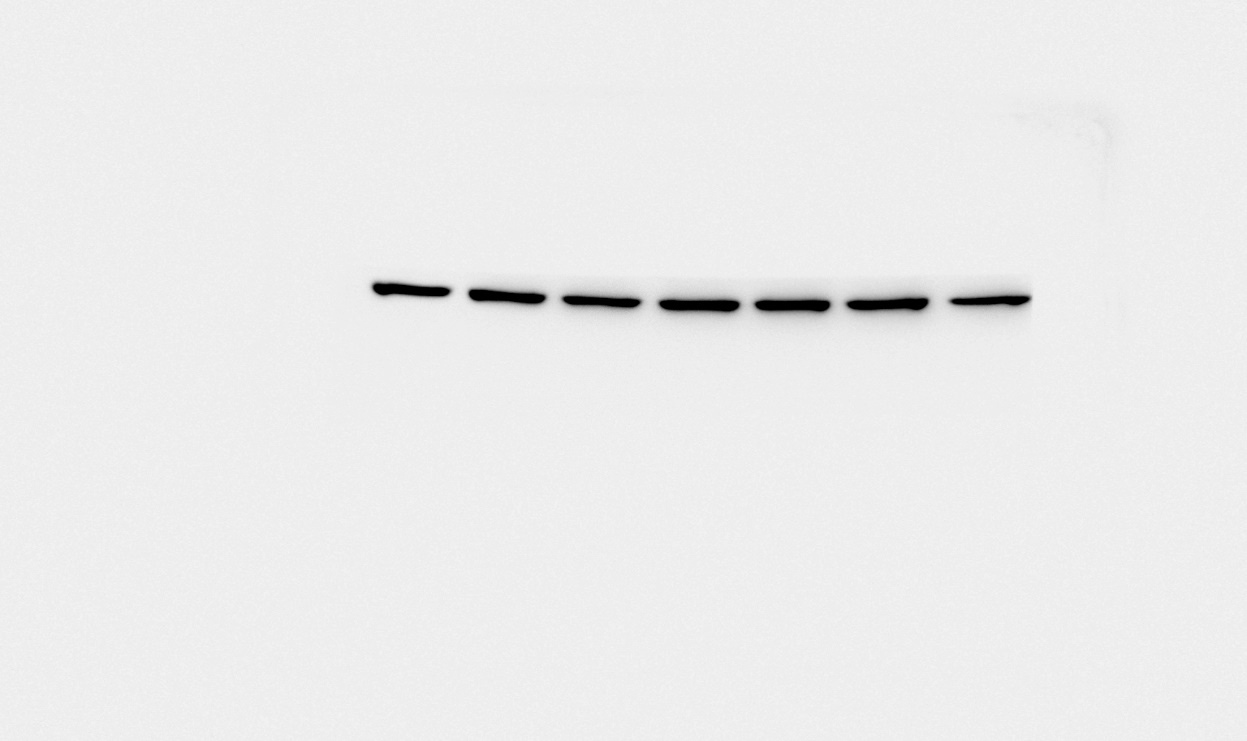  **20**  **48**  **35**  **25**  **β-actin**  **17** |

**Proteins from Cytoplasmic extraction**

**p-IκB-α**

| **p-IκB-α** | **A: Exposure time 1/100 sec** | **B: Exposure time 100 sec** |
| --- | --- | --- |
| **Exp.1** | 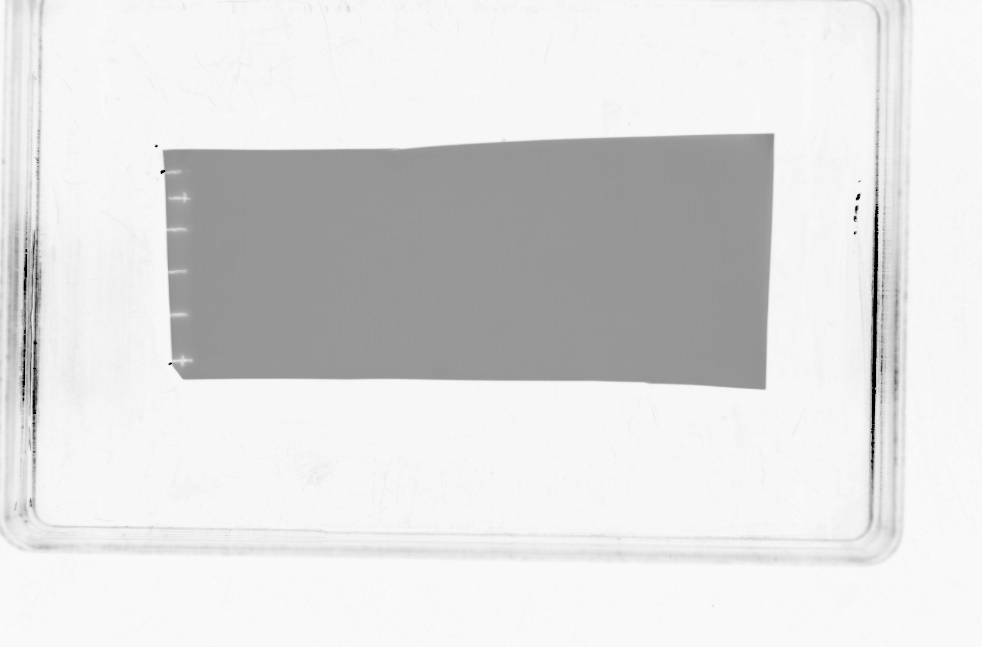  **75**  **48**  **35**  **25**  **63** 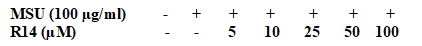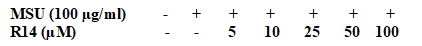 | 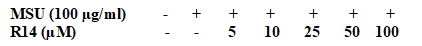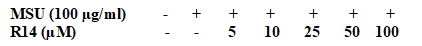 **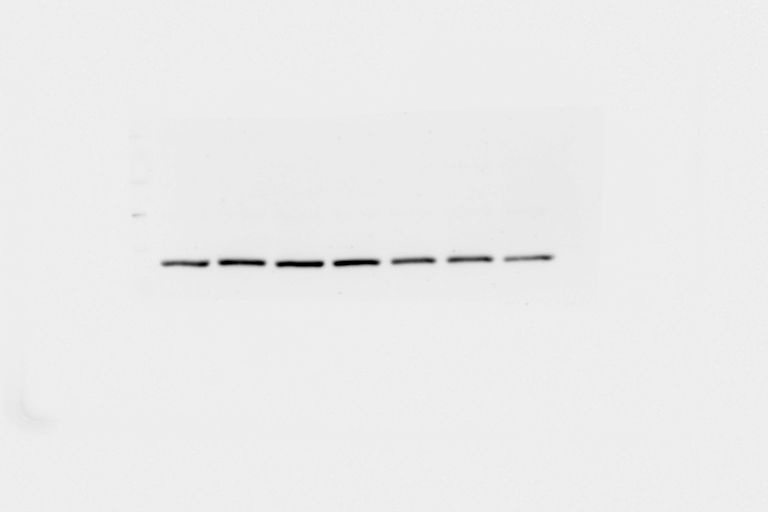**  **75**  **48**  **35**  **25**  **63**  **p-IκB-α** |
| **Exp.2** | **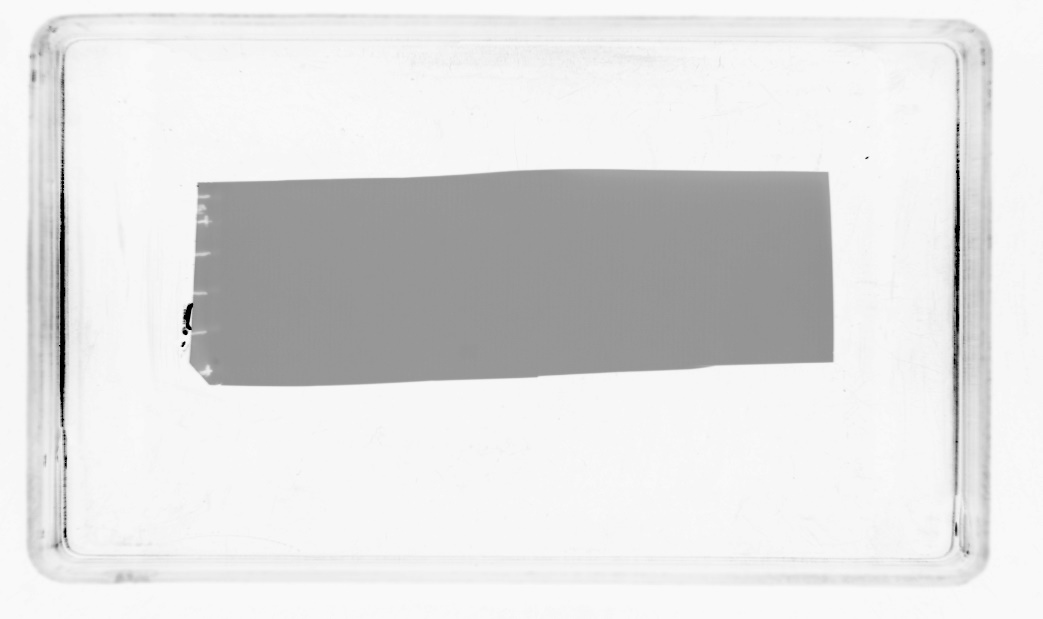**  **75**  **48**  **35**  **25**  **63** | **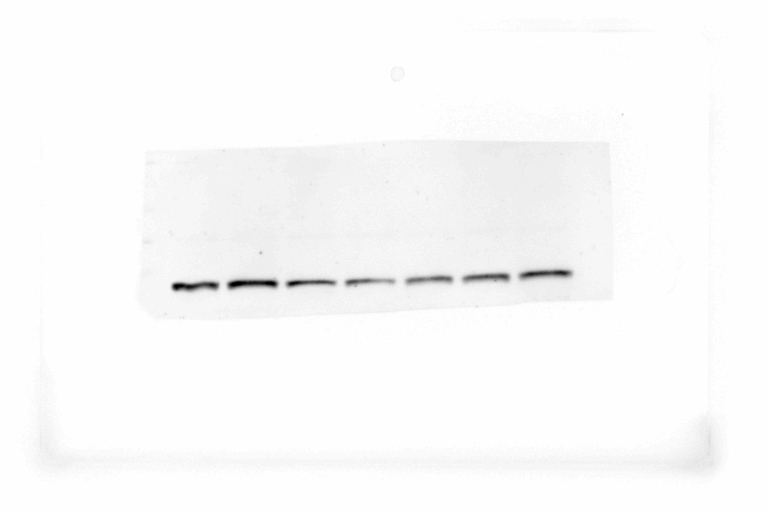**  **p-IκB-α** |
| **Exp.3** | **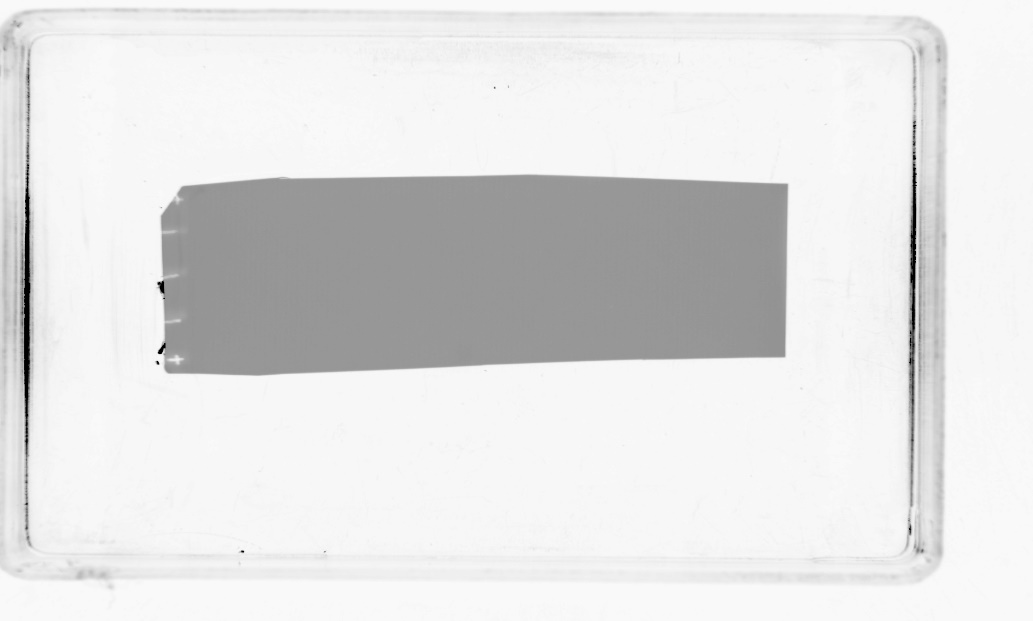**  **75**  **48**  **35**  **25**  **63** | **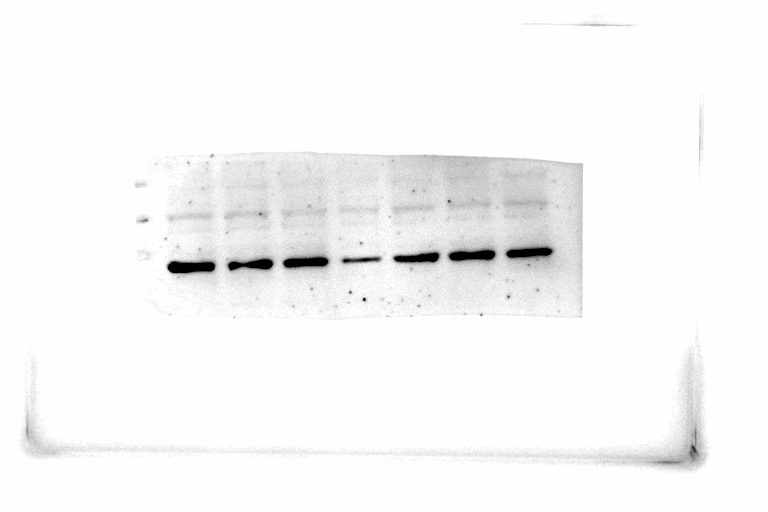**  **75**  **48**  **35**  **25**  **63**  **75**  **48**  **35**  **25**  **63**  **48** |

| **β-actin** | **A: Exposure time 1/100 sec** | **B: Exposure time 20 sec** |
| --- | --- | --- |
| **Exp.1** | **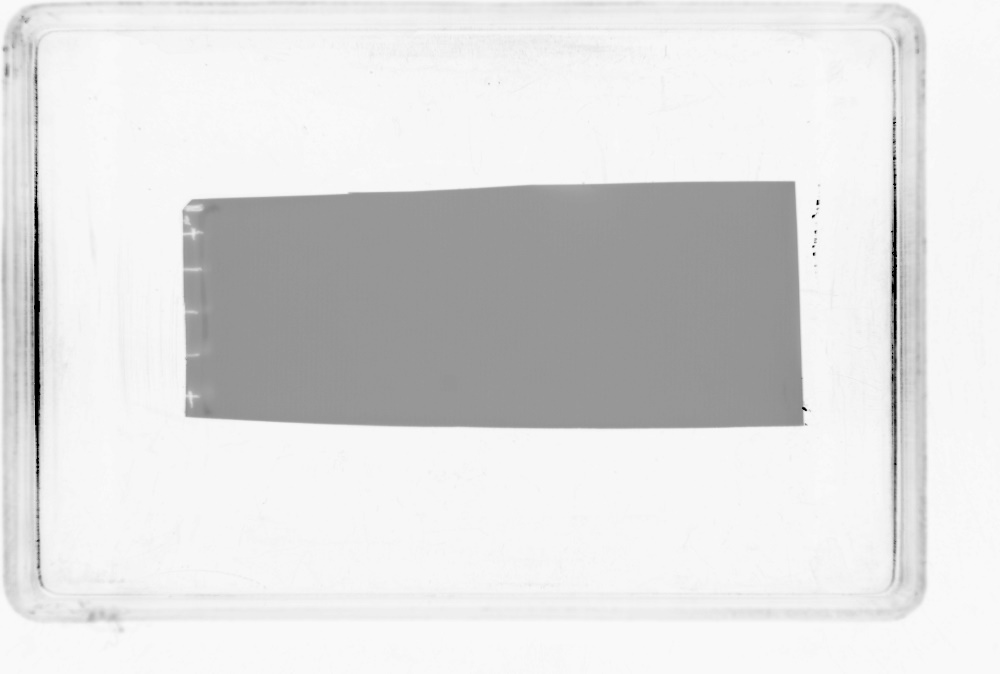**  **48**  **35**  **63**  **25** 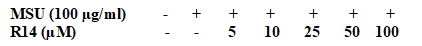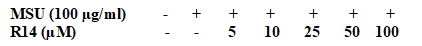 | **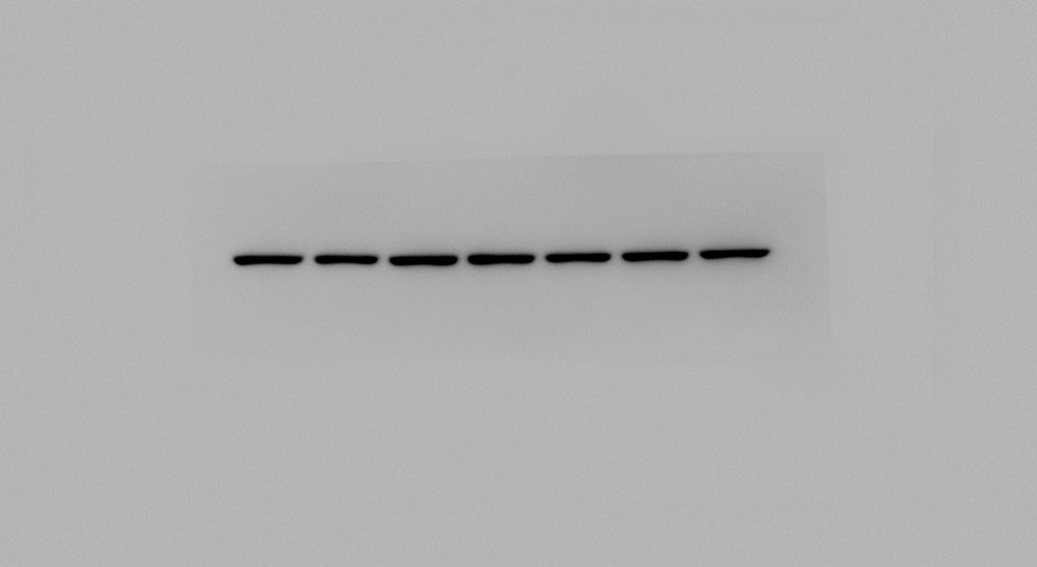**  **β-actin**  **48**  **35**  **63**  **25** 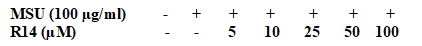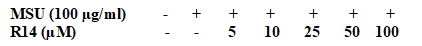 |
| **Exp.2** | **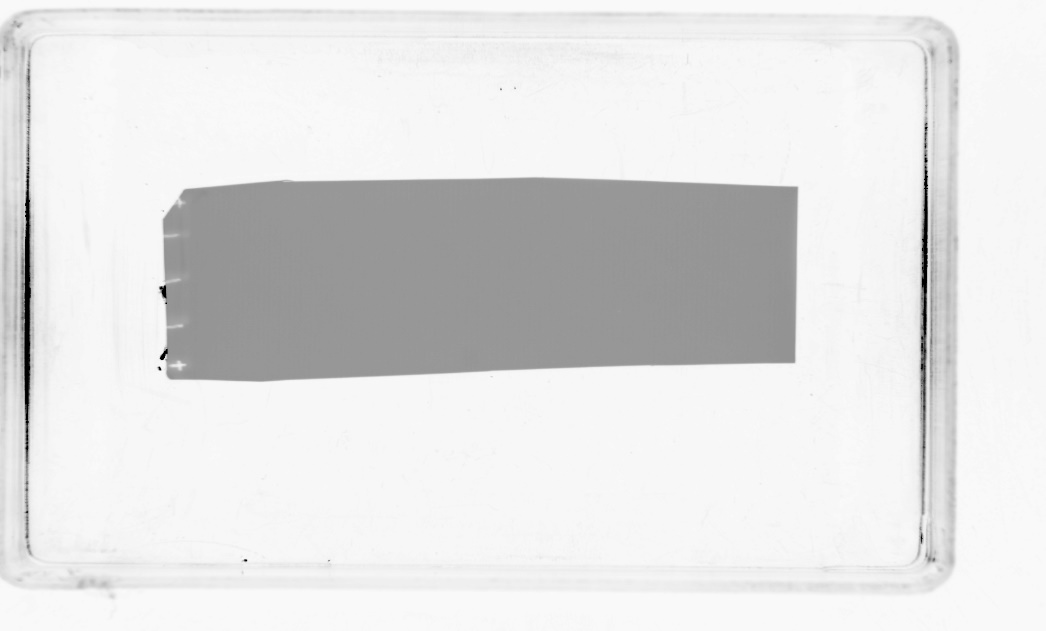**  **48**  **35**  **63**  **25** | 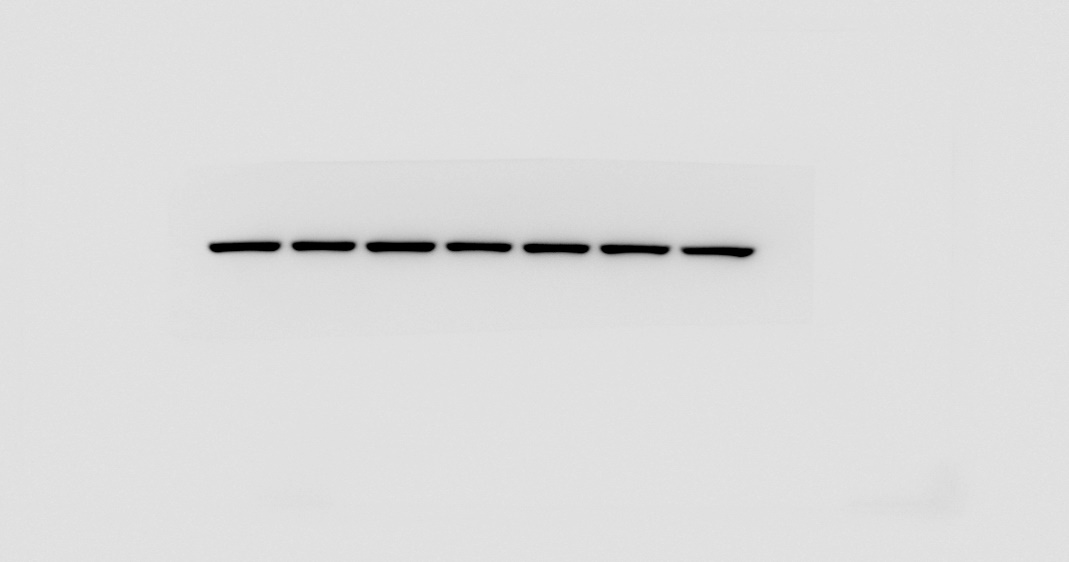  **β-actin**  **48**  **35**  **63**  **25** |
| **Exp.3** | **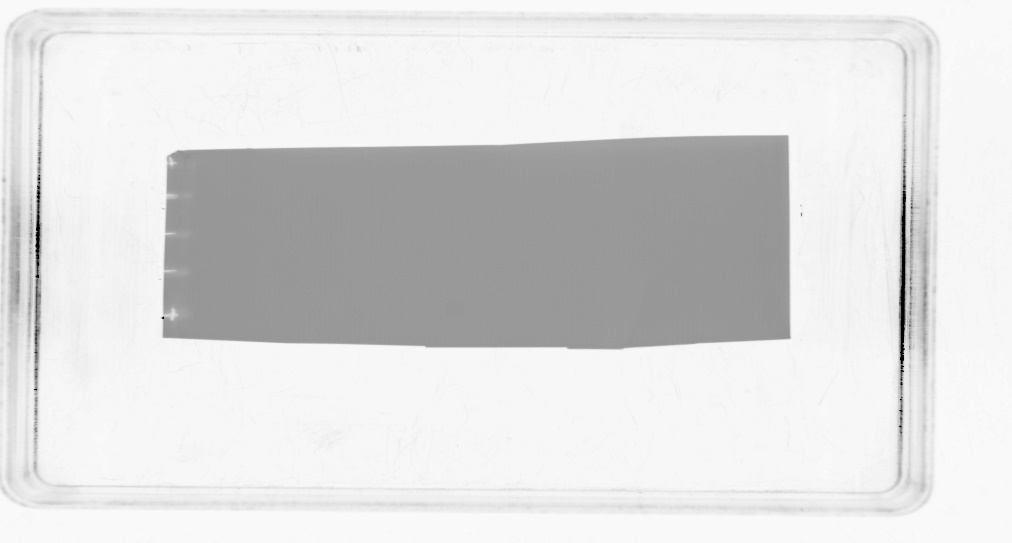**  **48**  **35**  **63**  **25** | **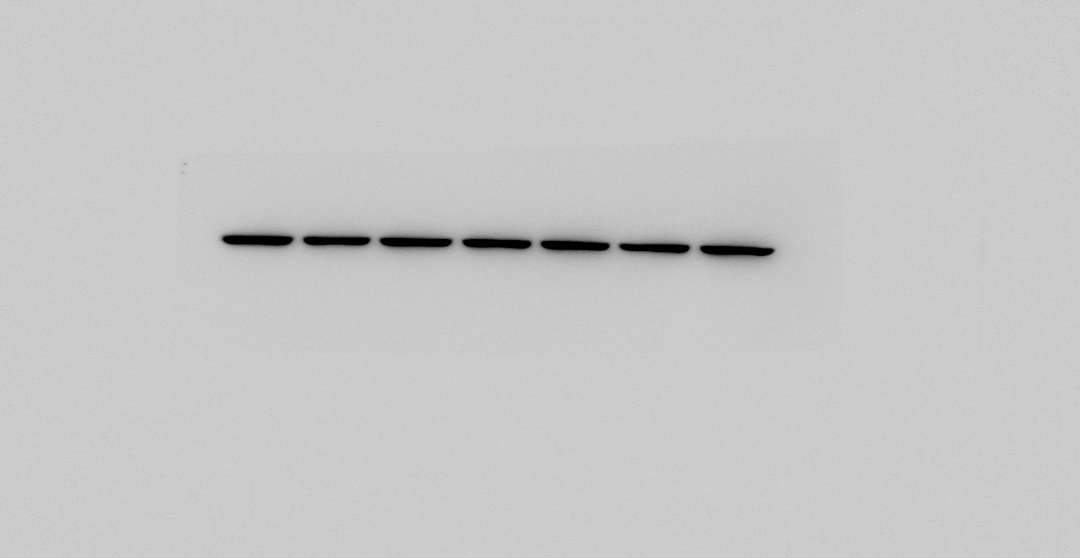**  **β-actin**  **48**  **35**  **63**  **25** |
